# Supplementary material for: Targeting dual-specificity tyrosine phosphorylation-regulated kinase 2 with a highly selective inhibitor for the treatment of prostate cancer
Source: Nat Commun. 2022 May 25;13:2903. doi: 10.1038/s41467-022-30581-4 (PMC9133015; doi:10.1038/s41467-022-30581-4)
Supplement: Supplementary file 1 — Supplementary Information [file 41467_2022_30581_MOESM1_ESM.pdf]

Supplementary Materials

Targeting dual-specificity tyrosine phosphorylation-regulated kinase 2 with a highly selective inhibitor for the treatment of prostate cancer

Kai Yuan[1,2,7], Zhaoxing Li[1,3,7], Wenbin Kuang[1,2,7], Xiao Wang[1,2,7], Minghui Ji[1,2], Weijiao Chen[1,2], Jiayu Ding[1,2], Jiaxing Li[1,2], Wenjian Min[1,2], Chengliang Sun[1,2], Xiuquan Ye[1,3], Meiling Lu[1,4], Liping Wang[1,2], Haixia Ge[5], Yuzhang Jiang[6,*], Haiping Hao[1,3,*], Yibei Xiao[1,3,*] and Peng Yang[1,2,*]

[1] State Key Laboratory of Natural Medicines and Jiangsu Key Laboratory of Drug Design and Optimization, China Pharmaceutical University, Nanjing 210009, China

[2] Department of Medicinal Chemistry, School of Pharmacy, China Pharmaceutical University, Nanjing 211198, China

[3] Department of Pharmacology, School of Pharmacy, China Pharmaceutical University, Nanjing 211198, China

[4] School of Life Science and Technology, China Pharmaceutical University, Nanjing 211198, China

[5] School of Life Sciences, Huzhou University, Huzhou 313000, China

[6] Department of Laboratory, Huai'an First People's Hospital, Nanjing Medical University, Huai'an, Jiangsu 223300, China

[7] These authors contributed equally

The PDF file includes:

Supplementary Figures S1 – S16

Supplementary Tables 1- 4

Supplementary Methods

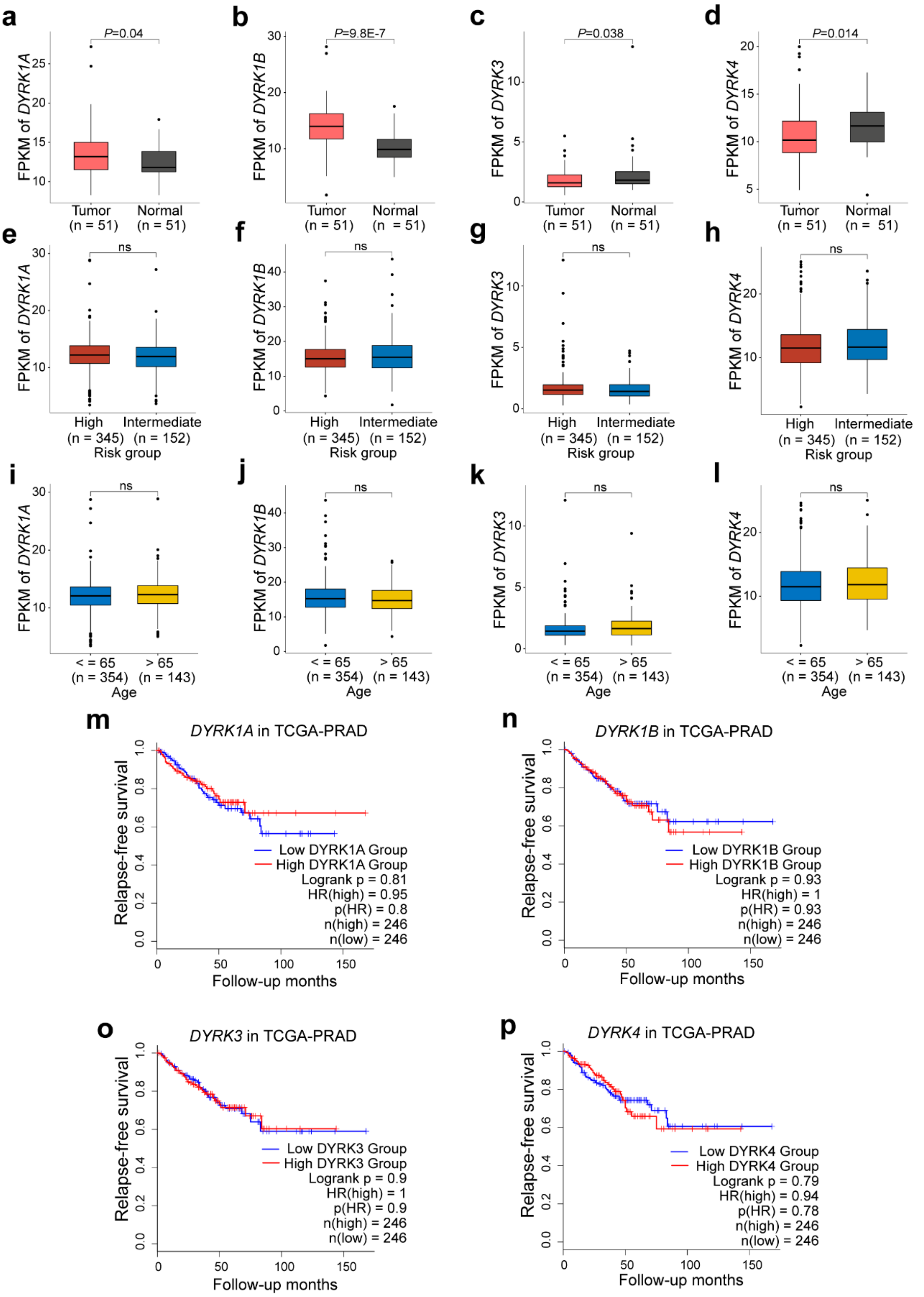

Supplementary Figure 1. Analysis of the expression of *DYRK1A*, *DYRK1B*, *DYRK3*, and *DYRK4* in prostate cancer.

a-d Comparison of *DYRK1A* (**a**), *DYRK1B* (**b**), *DYRK3* (**c**), and *DYRK4* (**d**) expression between tumor (red, n = 51) and normal (gray, n = 51) tissues. **e-h** Comparison of

DYRK1A (**e**), *DYRK1B* (**f**), *DYRK3* (**g**), and *DYRK4* (**h**) expression between high (red, n = 345) and intermediate (blue, n = 152) risk PCa patients. **i-l** Comparison of *DYRK1A* (**i**), *DYRK1B* (**j**), *DYRK3* (**k**), and *DYRK4* (**l**) expression between age <= 65 (blue, n = 354) and > 65 (yellow, n = 143) PCa patients. The whiskers of boxplot represent the quantile percentile, from bottom to top are minima, 25%, median, 75%, and maxima respectively. Two-tailed Student's *t* test was applied without adjustment for multiple comparisons (FDR). ns, not significant. **m-p** Kaplan-Meier survival plot of high (red line, n = 246) and low (blue line, n = 246) *DYRK1A* (**m**), *DYRK1B* (**n**), *DYRK3* (**o**), and *DYRK4* (**p**) expression PCa patients.

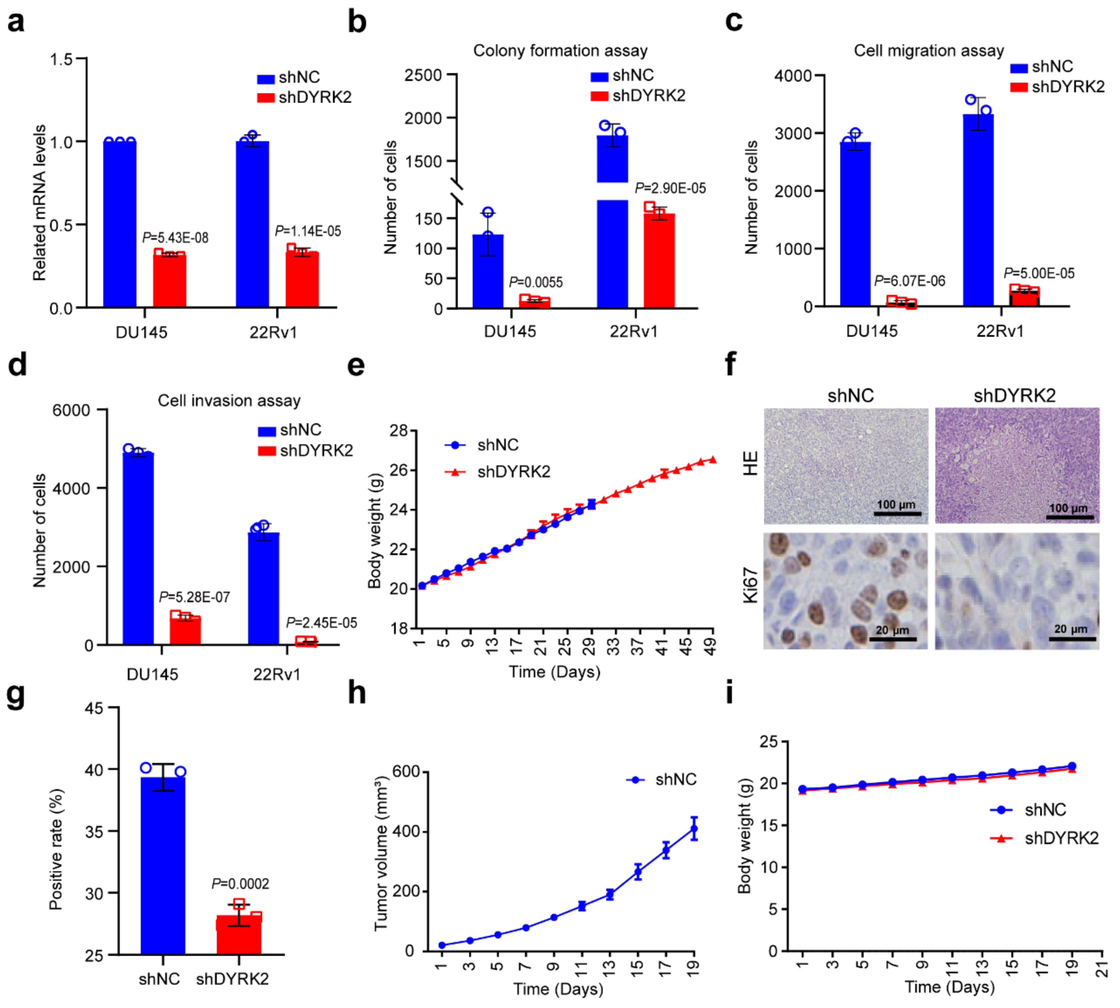

Supplementary Figure 2. DYRK2 KD significantly inhibited PCa.

a *DYRK2* mRNA level of in DU145 shNC/shDYRK2 cells and 22Rv1 shNC/shDYRK2 cells. Unpaired two-tailed Student's *t* test. Error bar, mean ± SD, n = 3. **b** Quantification of DU145 shNC/shDYRK2 cells and 22Rv1 shNC/shDYRK2 cells colony numbers after growth for ten days. Unpaired two-tailed Student's *t* test. Error bar, mean ± SD, n = 3. **c**, **d** Quantification of migration (**c**) and invasion (**d**) ability of DU145 shDYRK2/shNC cells and 22Rv1 shDYRK2/shNC cells. Unpaired two-tailed Student's *t* test. Error bar, mean ± SD, n = 3. **e**, **f** BALB/c nude mice were implanted subcutaneously with DU145 shNC (n = 6) and shDYRK2 (n = 10) cells. Body weight of mice (**e**) was measured every two days. The shNC group were euthanazied at 29th day and shDYRK2 group were euthanazied at 49th day. Representative images of H&E and Ki-67 (**f**) of paraffin section of tumor from mice treated with DU145 shDYRK2 and shNC cells. Error bar, mean ± SD. **g** Quantification of Ki67 positive rate of tumor

from mice treated with DU145 shNC and shDYRK2cells. Unpaired two-tailed Student's *t* test. Error bar, mean ± SD, n = 3. **h**, **i** BALB/c nude mice were implanted subcutaneously with 22Rv1 shNC (n = 6) and shDYRK2 (n = 8) cells. Tumor volume of mice (**h**) and Body weight of mice (**i**) were measured every two days. The mice were euthanatized at 19th day. Error bar, mean ± SD. Source data are provided as a Source Data file.

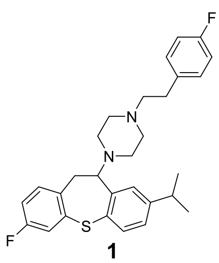

1

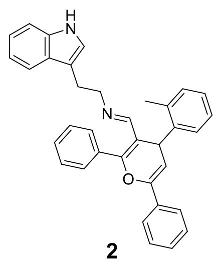

2

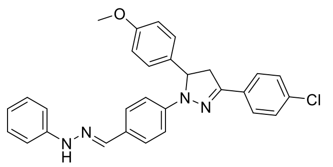

3

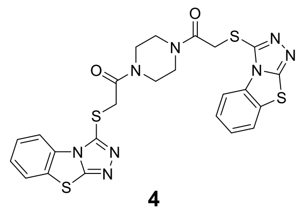

4

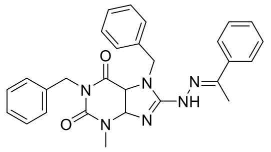

5

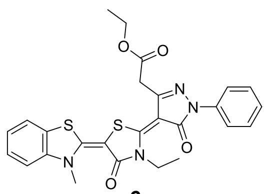

6

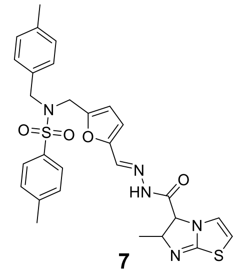

7

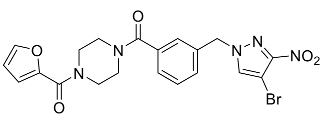

8

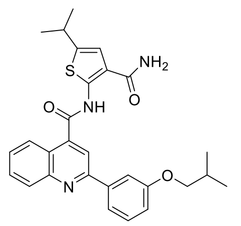

9

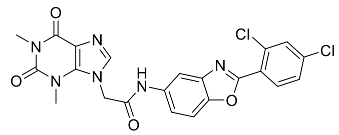

10

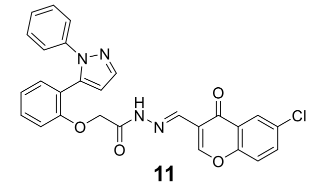

11

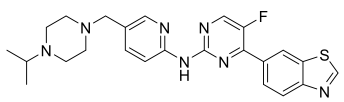

12

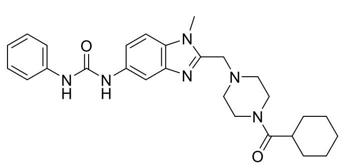

13

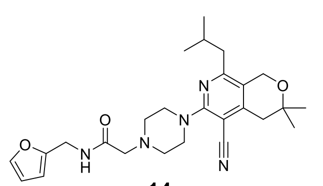

14

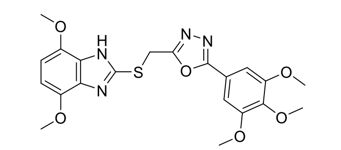

15

Supplementary Figure 3. Chemical structures of 15 selected compounds for kinase evaluation.

Compounds **1-15** were selected and further tested for their inhibitory activity against DYRK2 kinase.

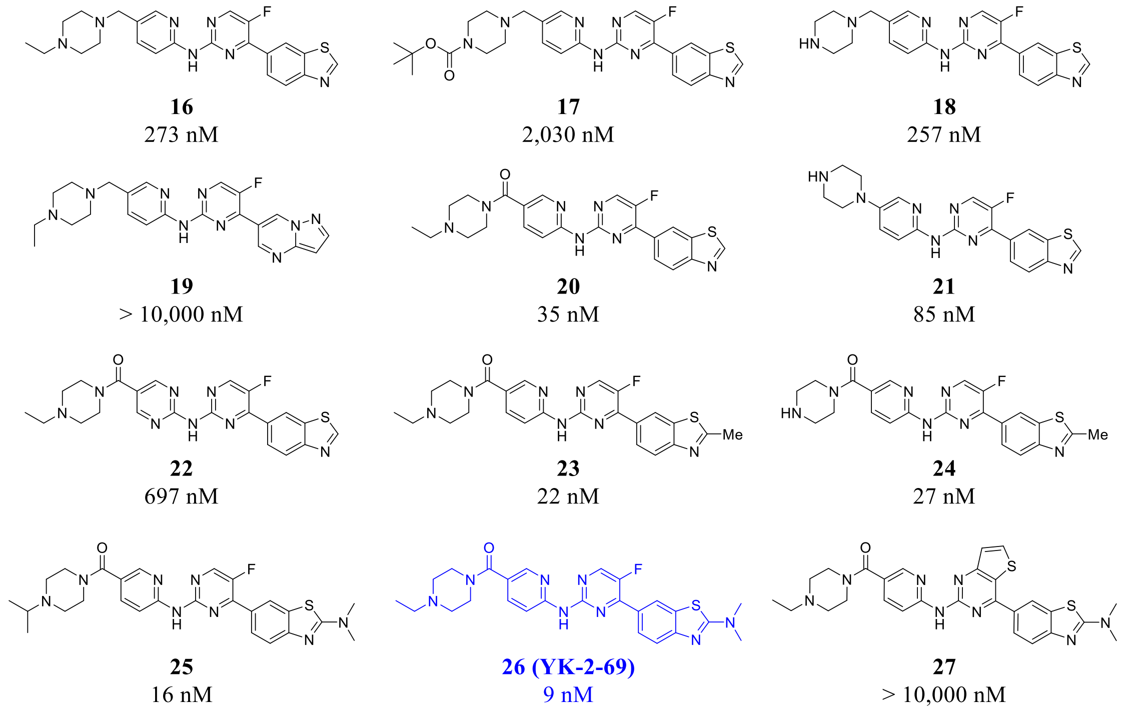

Supplementary Figure 4. Chemical structures and DYRK2 kinase IC_{50} values of compounds 16-27.

To further improve DYRK2 inhibitory activity, representative derivatives **16-27** were synthesized and further tested for their inhibitory activity against DYRK2 kinase. Among them, compound **26** (re-named as YK-2-69) exhibited the most potent DYRK2 inhibitory activity with an IC_{50} value of 9 nM. The IC_{50} Values are the means of duplicate experiments and calculated with GraphPad Prism 8. Source data are provided as a Source Data file.

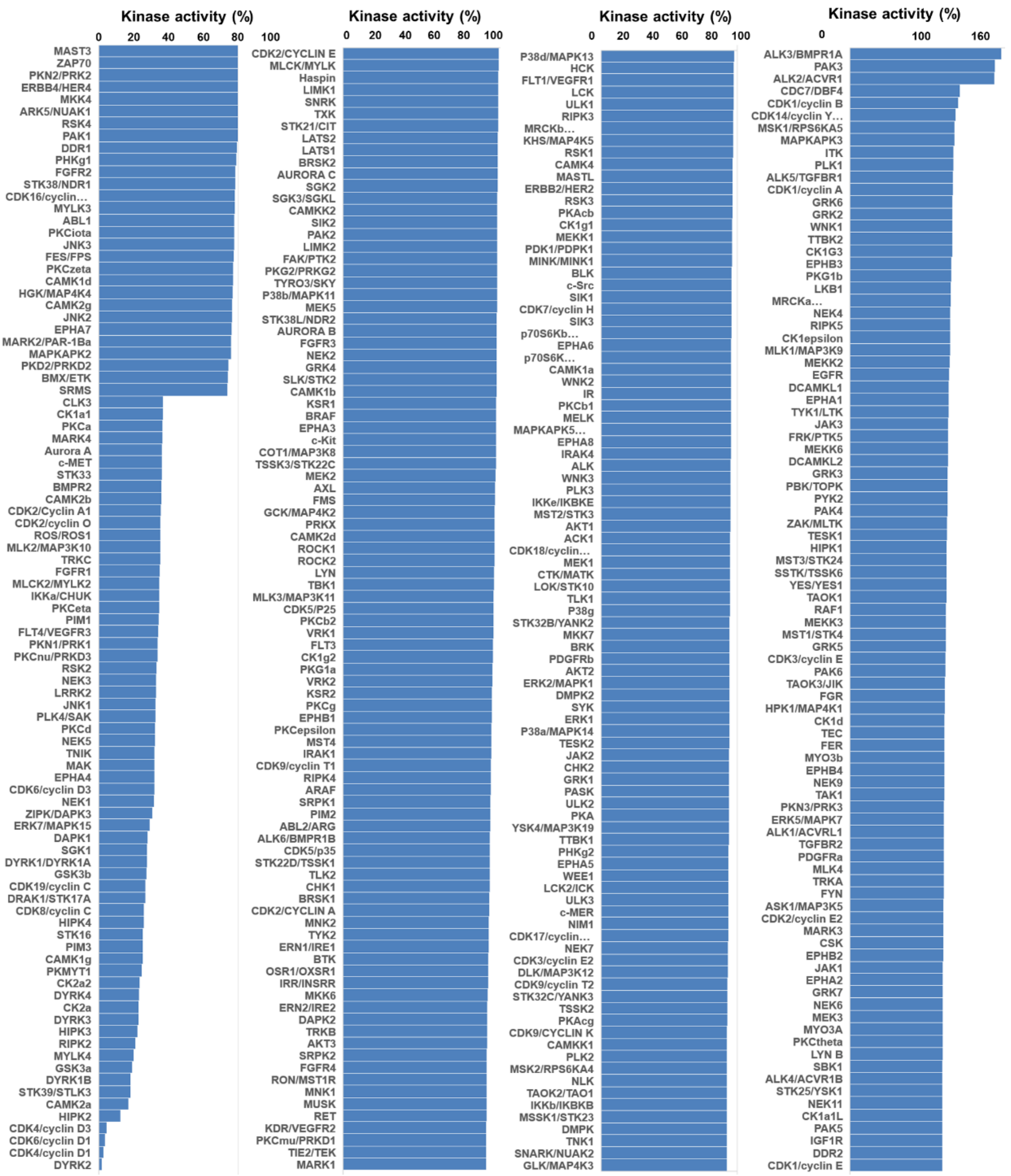

Supplementary Figure 5. YK-2-69 displayed great selectivity over 370 kinases. Kinase specificity of YK-2-69 was carried out at Reaction Biology Corporation (https://www.reactionbiology.com/). Inhibitory activity assays of YK-2-69 at the concentration of 1 μM against a panel of 370 kinases were conducted. Source data are provided as a Source Data file.

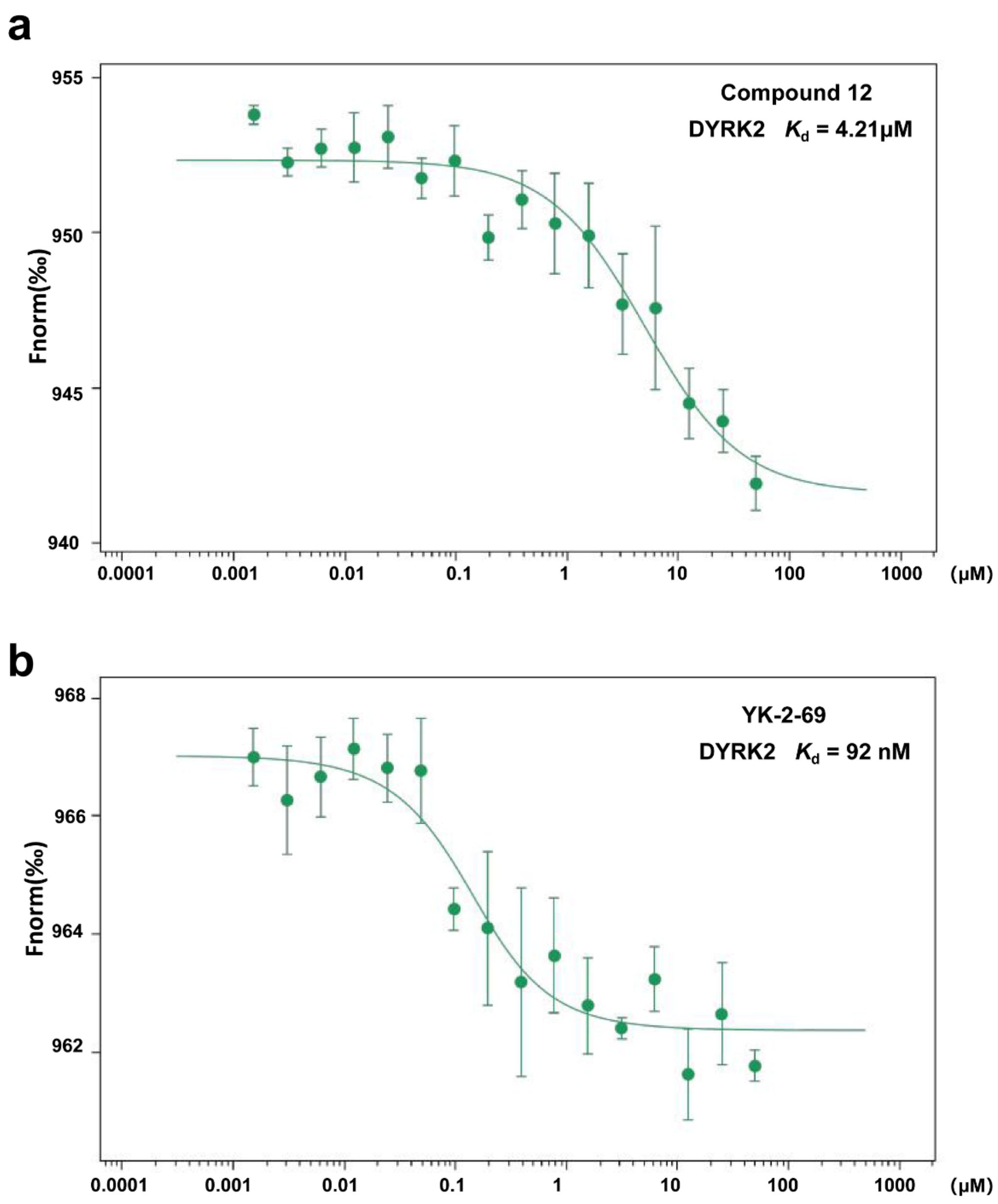

Supplementary Figure 6. The binding constants of compound 12 and YK-2-69 with DYRK2.

a, **b** The binding constants of compound **12** (**a**) and YK-2-69 (**b**) with DYRK2 were determined by microscale thermophoresis assay. Error bar, mean ± SD, n = 3. Source data are provided as a Source Data file.

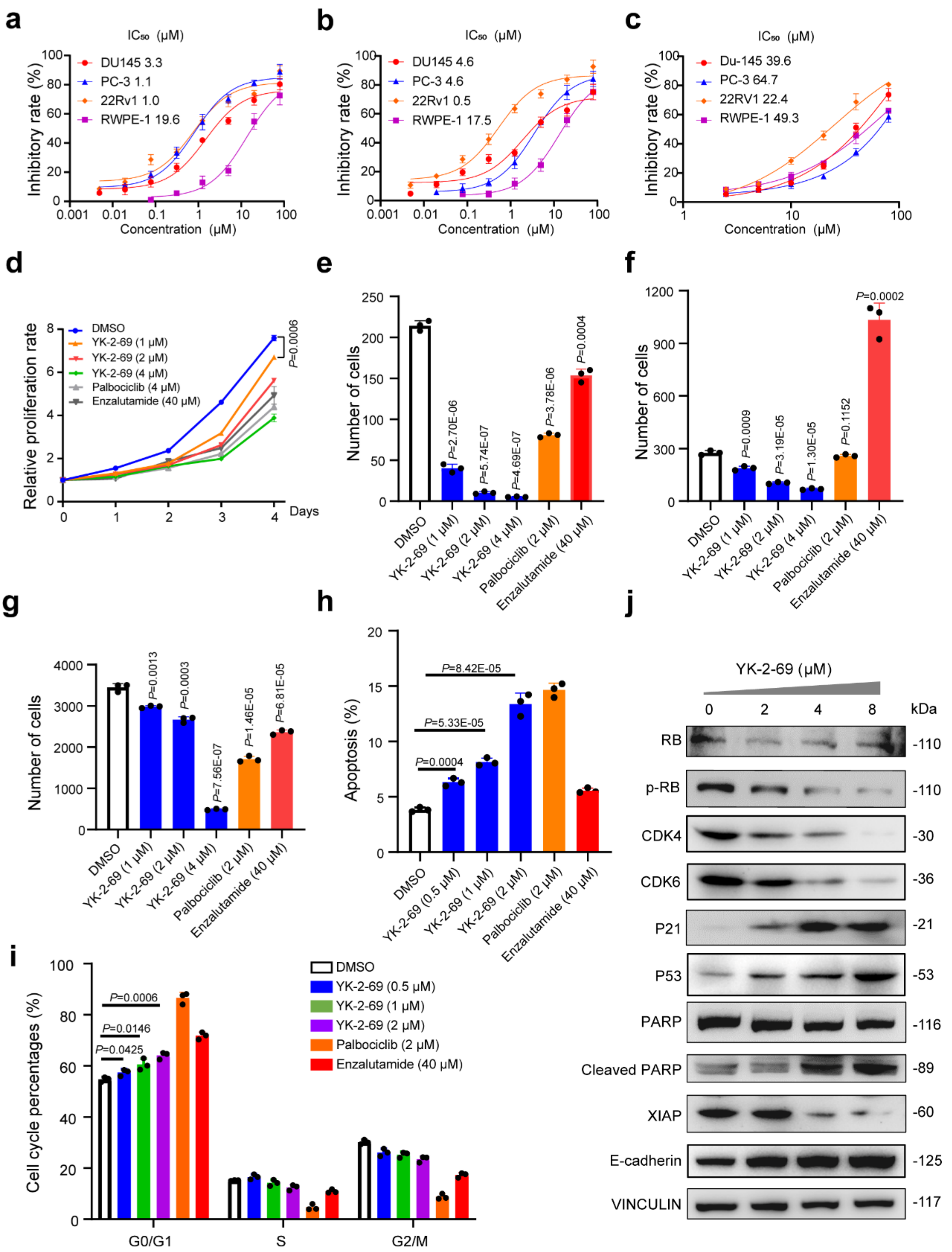

Supplementary Figure 7. YK-2-69 suppressed cell proliferation and metastasis and promoted apoptosis in PCa cells.

a-c Antiproliferative activities of YK-2-69 (**a**), palbociclib (**b**) and enzalutamide (**c**) against of DU145, PC-3, 22Rv1, and RWPE-1 cell lines. Error bar, mean ± SD, n = 3. **d** Effects of YK-2-69 (1, 2, 4 μM), palbociclib (4 μM), or enzalutamide (40 μM) treatment on the viability of the PC-3 cells during a 5-day course. Unpaired two-tailed

Student's *t* test. Error bar, mean ± SD, n = 3. **e** Quantification of PC-3 cells colony numbers. Before being plated on the 24-well plate for colony formation, PC-3 cells were treated with DMSO, YK-2-69 (1, 2, 4 μM), palbociclib (2 μM), or enzalutamide (40 μM) for 48 h. Unpaired two-tailed Student's *t* test. Error bar, mean ± SD, n = 3. **f**, **g** Quantification of migration (**f**) and invasion (**g**) ability of PC-3 after treatment with DMSO, YK-2-69(1, 2, 4 μM), palbociclib (2 μM), or enzalutamide (40) for 48 h. Unpaired two-tailed Student's *t* test. Error bar, mean ± SD, n = 3. **h** Apoptosis of PC-3 cells after treatment with DMSO, YK-2-69 (0.5, 1, 2 μM), palbociclib (2 μM), or enzalutamide (40 μM) for 48 h determined by flow cytometry. Unpaired two-tailed Student's *t* test. Error bar, mean ± SD, n = 3. **i** Cell cycle phase distribution of PC-3 cells after treatment with DMSO, YK-2-69 (0.5, 1, 2 μM), palbociclib (2 μM), or enzalutamide (40 μM) for 48 h determined by flow cytometry. Unpaired two-tailed Student's *t* test. Error bar, mean ± SD, n = 3. **j** Western blotting analysis of indicated proteins in DU145 cells after treatment with DMSO or YK-2-69 (2, 4, 8 μM) for 48 h. Source data are provided as a Source Data file.

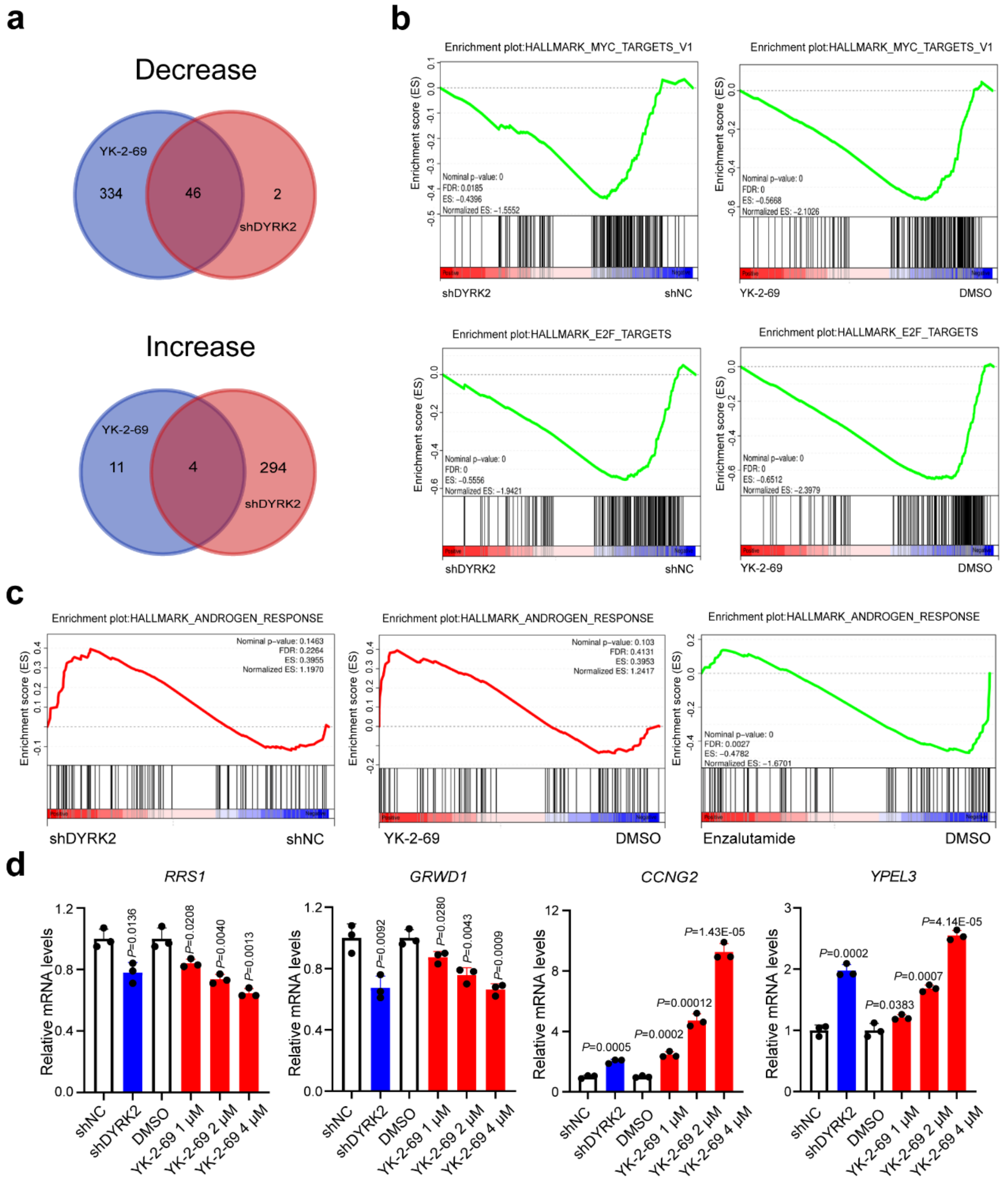

Supplementary Figure 8. Transcriptome-wide RNA sequencing assays in PCa 22Rv1 cells.

a Transcriptome strategy of RNA-sequencing conducted on 22Rv1 cells exposed to YK-2-69 (1 μM) or enzalutamide (20 μM) for 48 h. The shNC, shDYRK2, DMSO, YK-2-69, and enzalutamide groups all contain two biological replicates. Venn diagram of upregulated and downregulated signaling pathways in DYRK2 KD- and YK-2-69-treated 22Rv1 cells. The number of genes in every signaling pathway is more than 50. Normalized enrichment score (NES) > 1 or < -1; p < 0.05; FDR < 0.25. **b** The signaling

pathways enriched in different groups obtained through Gene set enrichment analysis (GSEA). **c** GSEA was used to analyze the effects of DYRK2 KD, YK-2-69 or enzalutamide treatment on the ANDROGEN RESPONSE signaling pathway in 22Rv1 cells. **d** Effects of DYRK2 KD or YK-2-69 treatment on the *RRS1*, *GRWD1*, *CCNG2*, and *YPEL3* mRNA levels in 22Rv1 cells. Unpaired Student's t test. Error bars, mean ± SD, n = 3. Source data are provided as a Source Data file.

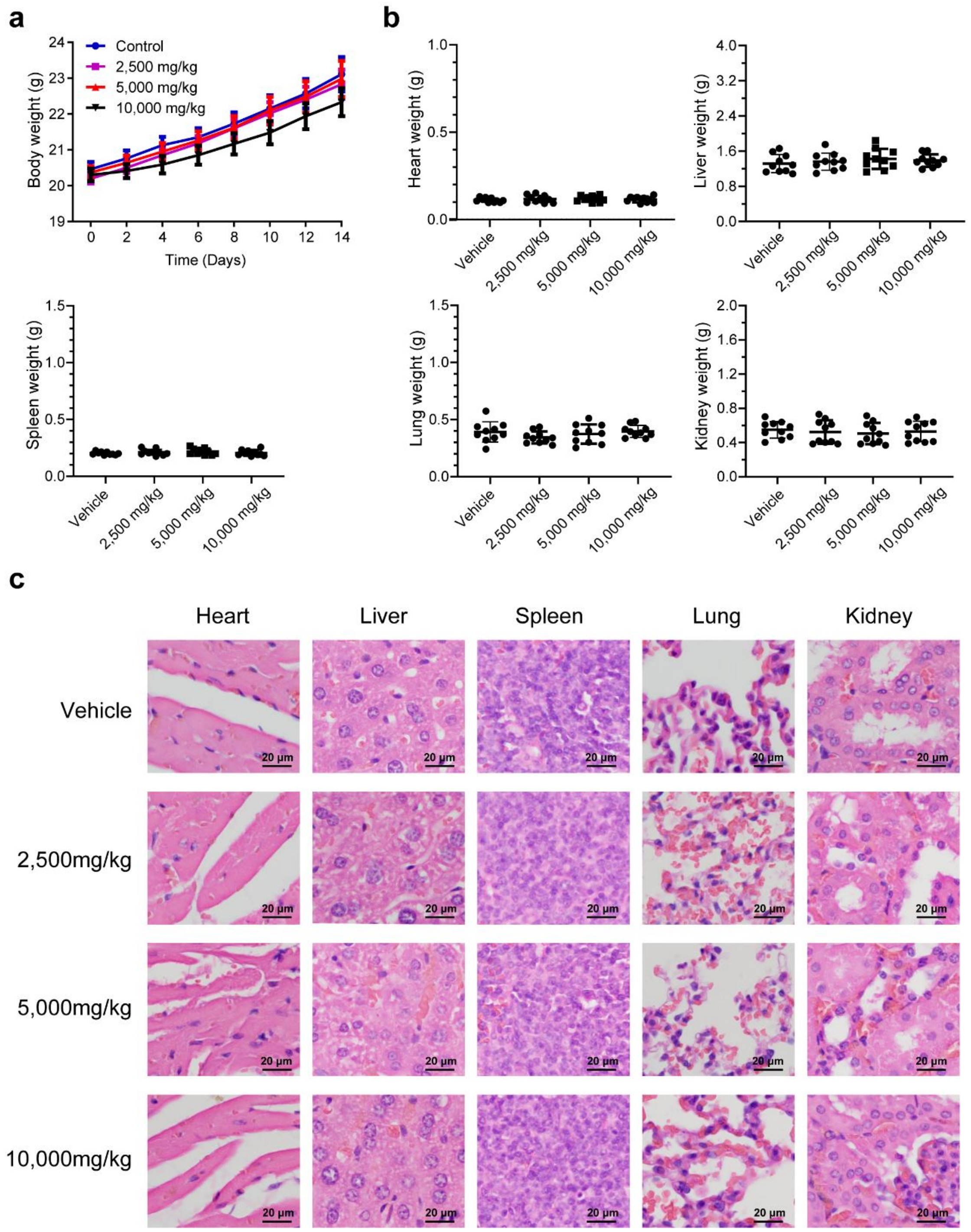

Supplementary Figure 9. YK-2-69 exhibited great safety properties *in vivo*.

a, **b** Mice were given vehicle or YK-2-69 (2,500 mg/kg, 5,000 mg/kg, and 10,000 mg/kg) orally. After one dose, the body weight of mice was measured every two days (**a**). After 14 days, no mice died, and all mice were executed and dissected. The main organs of mice, including the heart, liver, spleen, lung, and kidney, were taken out and weighed (**b**). Error bars, mean ± SD, n = 10. **c** Representative images of H&E staining of main organs from mice treated with vehicle or YK-2-69 (2,500 mg/kg, 5,000 mg/kg,

and 10,000 mg/kg). Source data are provided as a Source Data file.

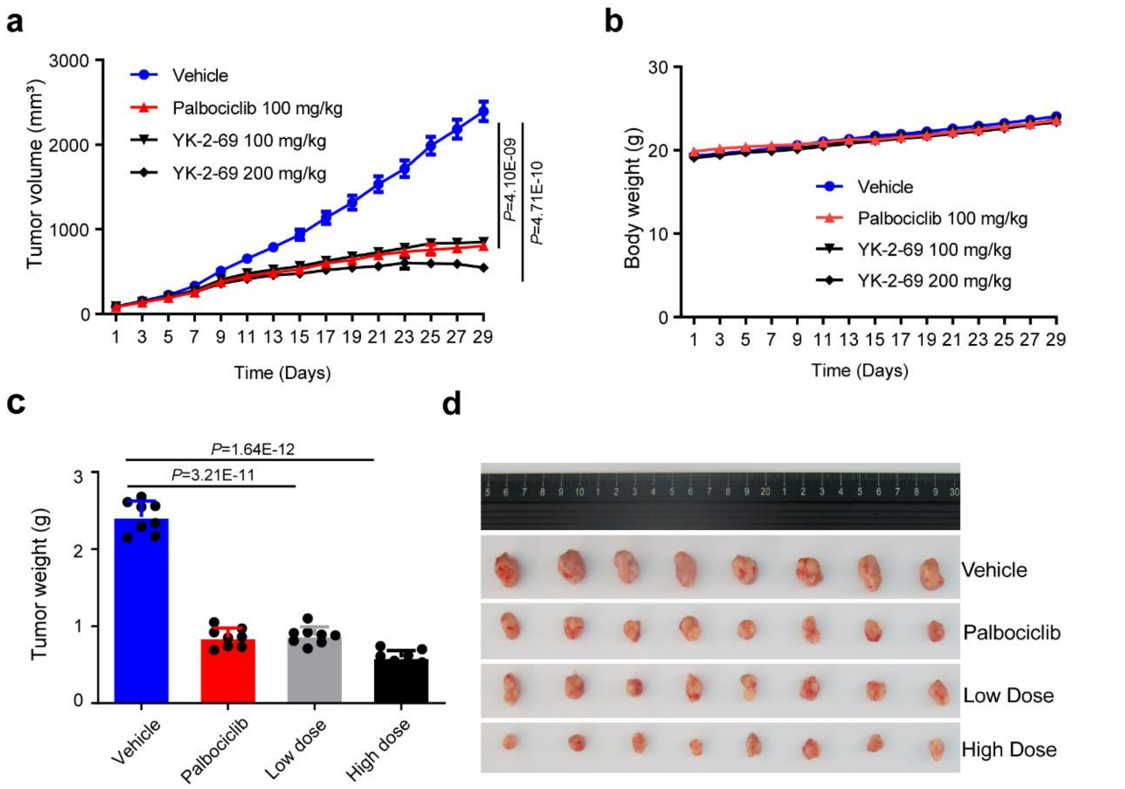

Supplementary Figure 10. YK-2-69 significantly inhibited tumor growth *in vivo*.

a-**d** BALB/c nude mice received subcutaneous injection of 1 × 10^7 PC-3 cells in the right flank. When tumors grew about 80-100 mm^3, four group mice (n = 8/group) were orally administrated vehicle, palbociclib (100 mg/kg), and YK-2-69 (100 and 200 mg/kg) every day, respectively. Tumor volumes (**a**) and body weight of mice (**b**) were measured every two days. After 29 days, mice were sacrificed. Tumor tissues of each group were weighed (**c**) and then photographed (**d**). Unpaired two-tailed Student's *t* test. Error bar, mean ± SD, n = 8. Source data are provided as a Source Data file.

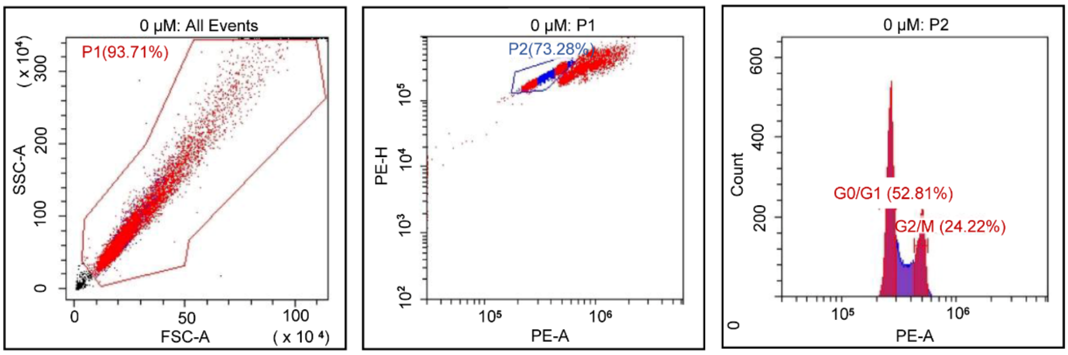

Supplementary Figure 11. The gating strategy in analyzing the results of cell cycle assays.

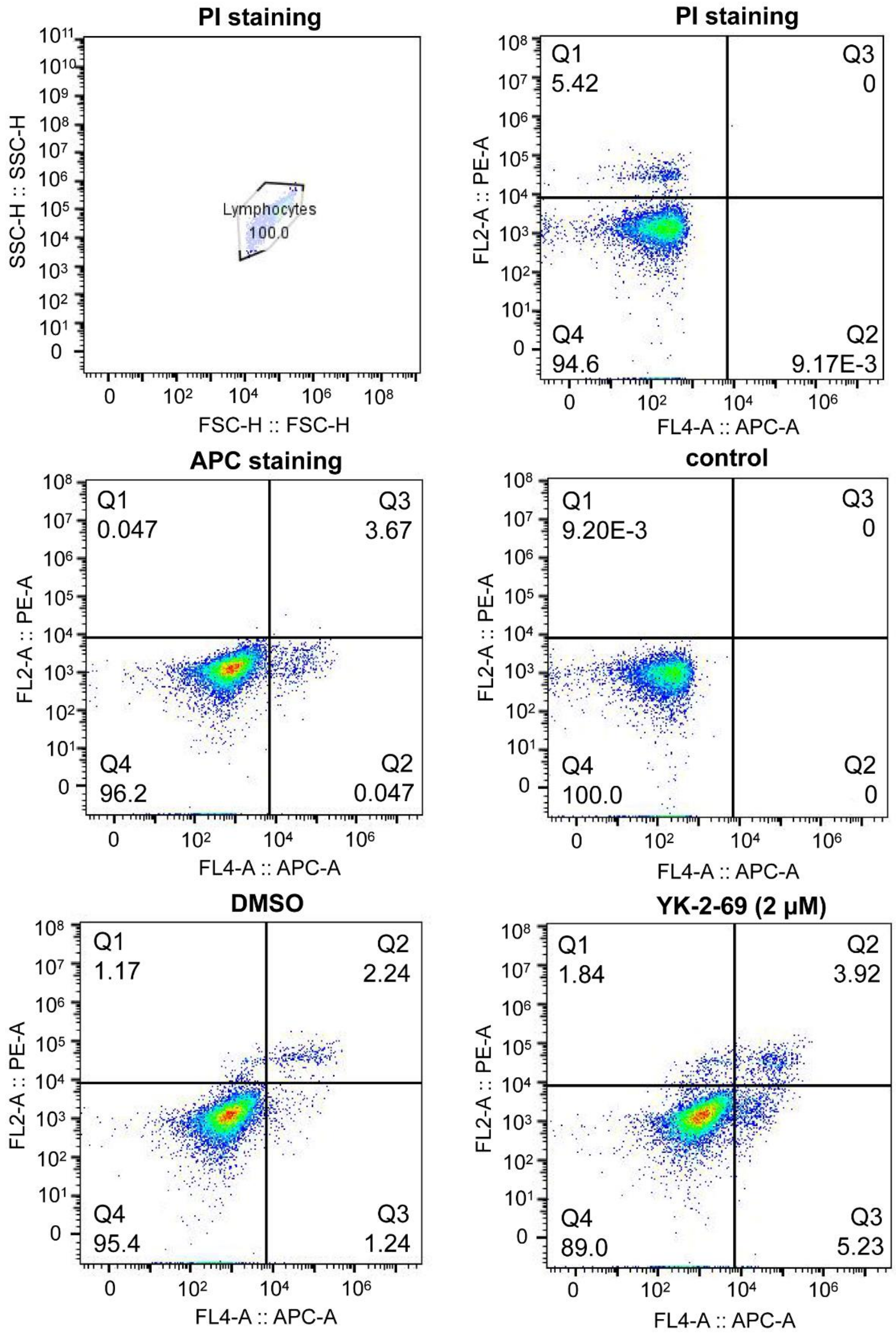

Supplementary Figure 12. The gating strategy in analyzing the results of cell apoptosis assays.

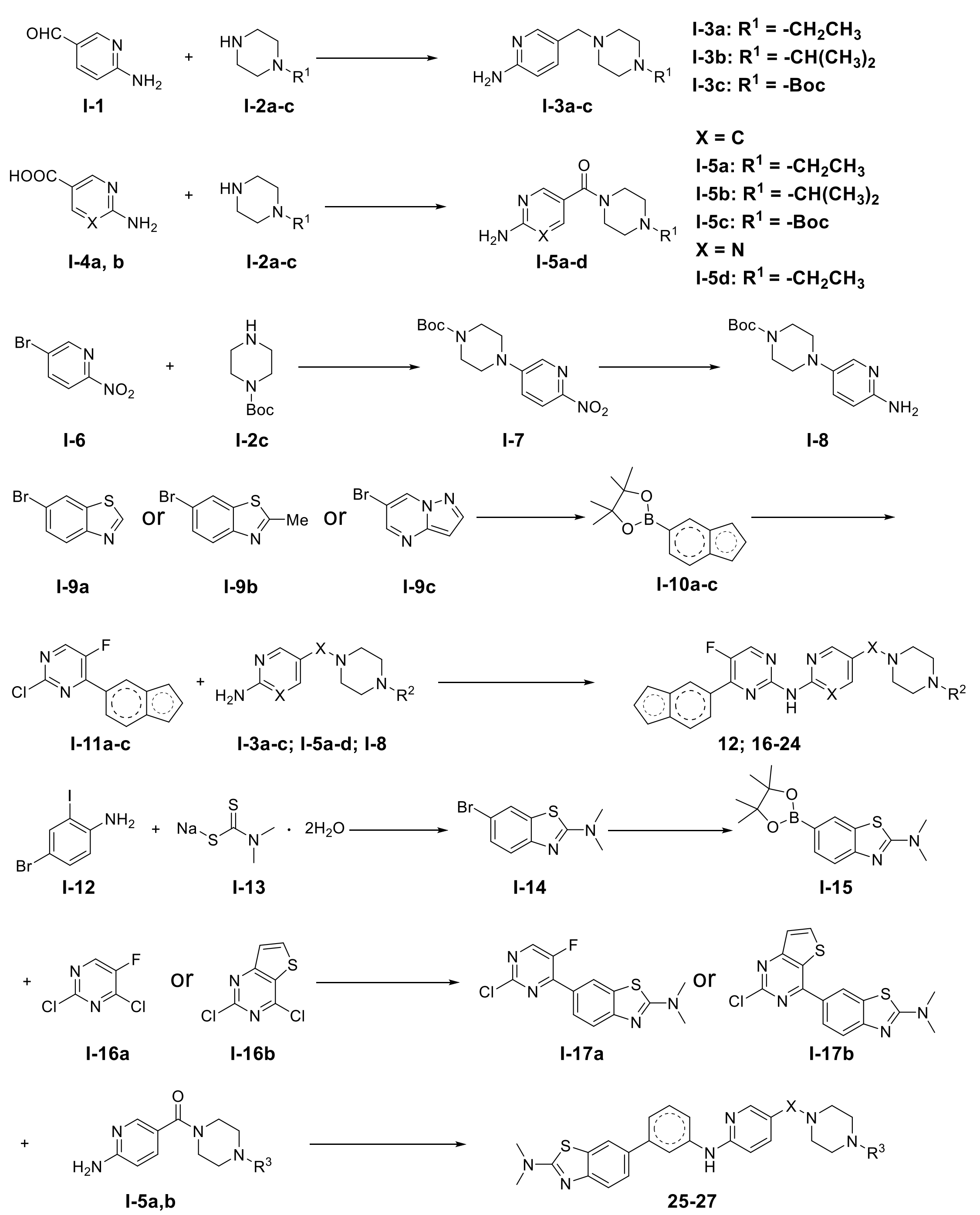

Supplementary Figure 13. General synthesis routes of compounds 16-27

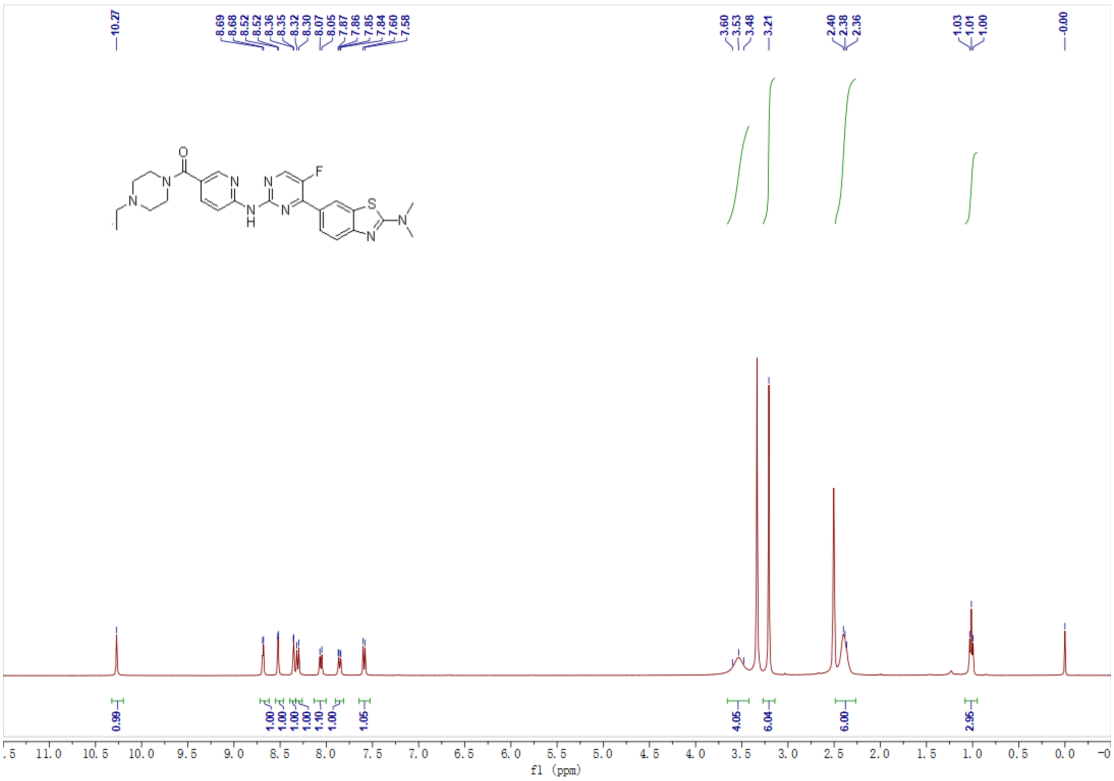

Supplementary Figure 14. ^{1}H NMR spectrum of YK-2-69.

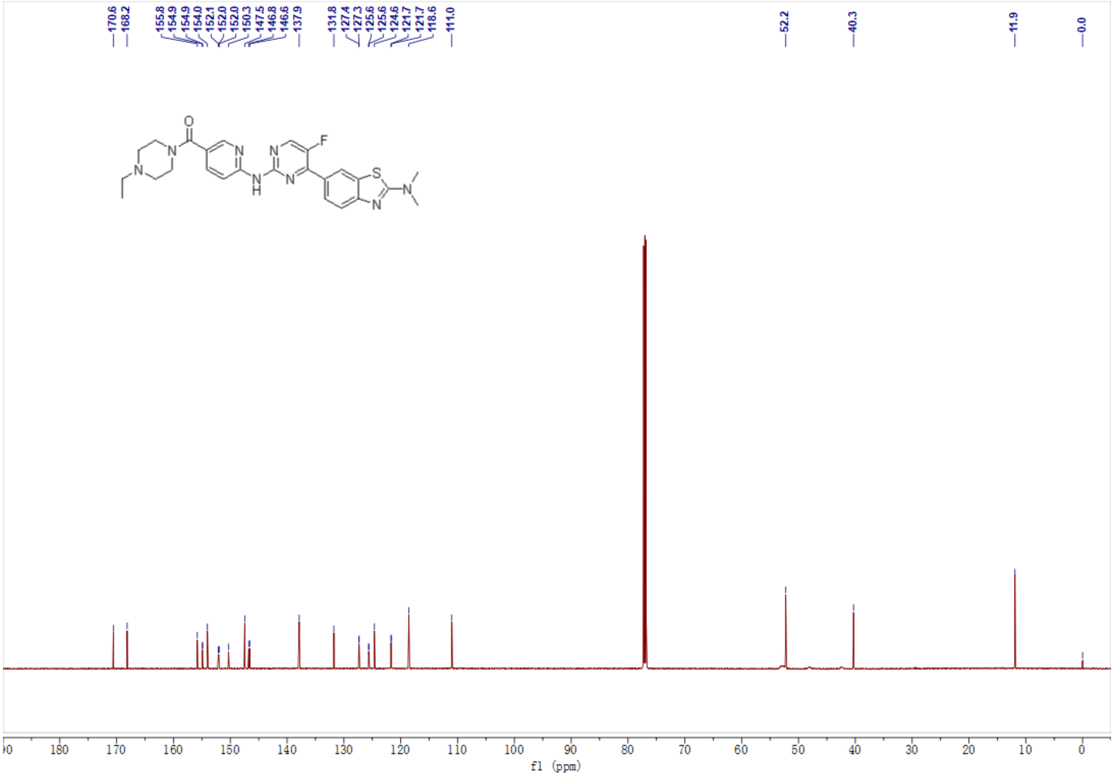

Supplementary Figure 15. ^{13}C NMR spectrum of YK-2-69.

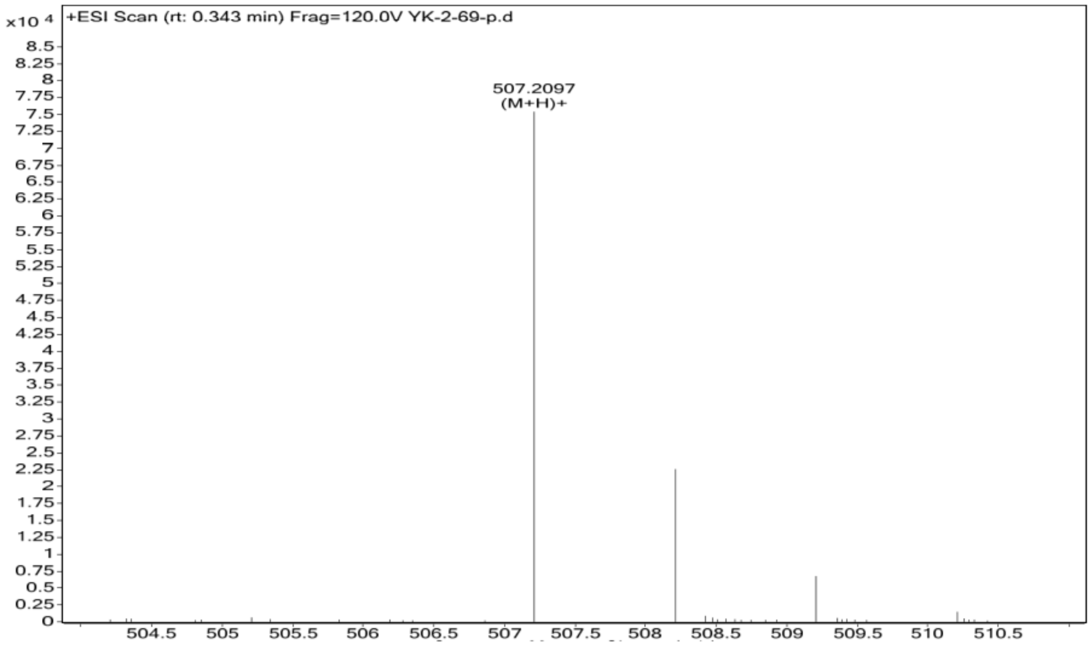

Supplementary Figure 16. HRMS of YK-2-69

Supplementary Table 1. Virtual screening and biological assay of 15 candidate hits[a]

No	Score 1	Score 2	MW	LogP	H Donors/ Acceptors	IC_{50} (μM)
**1**	127.32	56.92	478.64	7.59	0/2	> 10
**2**	140.06	56.22	494.62	7.72	1/3	> 10
**3**	131.23	61.67	480.99	7.45	1/5	> 10
**4**	141.63	55.19	580.73	4.26	0/10	> 10
**5**	132.21	57.18	478.54	5.21	1/8	> 10
**6**	135.98	56.50	520.62	4.52	0/8	> 10
**7**	146.32	57.95	561.68	4.60	1/9	> 10
**8**	131.01	57.47	488.29	2.93	0/10	> 10
**9**	126.54	55.25	487.61	5.99	3/6	> 10
**10**	136.95	61.37	499.31	3.04	1/10	> 10
**11**	130.86	55.06	498.92	4.63	1/8	> 10
**12**	140.30	63.44	463.58	4.50	1/7	0.263
**13**	128.06	58.36	474.60	3.86	2/8	> 10
**14**	125.14	55.49	465.59	3.16	1/8	> 10
**15**	125.94	55.69	458.49	3.42	1/10	> 10
**Staurosporine**						0.179

[a]Score 1: Libdock Score; Score 2: -CDOCKER INTERACTION ENERGY; MW: Molecular Weight; H Donors/ Acceptors: Number of hydrogen bond donors and acceptors in Lipinski rule; Staurosporine is the positive control in DYRK2 kinase assays. Source data are provided as a Source Data file.

Supplementary Table 2. Co-crystal data collection, phasing and refinement statistics

	DYRK2-YK-2-69
**Data Collection**	
Beam Line	BL19U, SSRF
Space group	C 2 2 21
Cell dimensions	
a, b, c (Å)	60.86 129.387 291.733
α, β, γ (°)	90 90 90
wavelength	0.9785
Resolution Limits (Å)	19.86 – 2.5 (2.589 – 2.5)
No. Unique reflections	40358 (3967)
Completeness (%)	99.70 (99.97)
CC1/2	0.998 (0.696)
R_{merge}	0.1797 (1.907)
$I/\sigma I$	14.02 (1.44)
Completeness (%)	99.70 (99.97)
**Refinement**	
No. reflections	40351
R_{work} / R_{free}	0.2115 / 0.2517
R.m.s.d for bonds (Å)	0.010
R.m.s.d for angles (°)	1.31
Number of non-hydrogen atoms	6351
Macromolecules	6279
Ligands	72
Solvent	0
B-factor	
Averaged	70.35
Macromolecules	70.44
Ligands	62.4
**Ramachandran plot (%)**	
Favored (%)	96.47
Allowed (%)	3.53
Outliers	0.00

Statistics for the highest-resolution shell are shown in parentheses.

Supplementary Table 3. Clinicopathologic information of three PCa patients

No.	Patient ID	Gender	Age	Gleason score	Seminal vesicle invasion	Bladder invasion	Lymph node metastasis
**1**	2019117695	Male	66	4 + 3 = 7	Yes	No	No
**2**	2019117753	Male	63	4 + 3 = 7	Yes	No	No
**3**	2019117934	Male	71	4 + 3 = 7	No	No	No

Supplementary Table 4. Primers of RT-qPCR reactions

	Forward (5′-3′)	Reverse (5′-3′)
DYRK2	GGGGAGAAAACGTCAGTGAA	TCTGCGCCAAATTAGTCCTC
RRS1	CCGAAAAGGGGTTGAAACTTCC	CCCTACCGGACACCAGAGTAA
GRWD1	GTGAGGGCTTTGCTCTTGAC	CACTGCAGATCCTCCACAGA
CCNG2	CTTTGGGCATTATTAGGA	GAGGAGGAAACAGTAGCAG
YPEL3	CAAACATCCAGACATGGTGAAG	CGGTGACAGCAAGTTAAATACAAA
GAPDH	ACCCAGAAGACTGTGGATGG	TTCAGCTCAGGGATGACCTT

Supplementary Methods

Structure-based virtual screening. Specs database (http://www.specs.net) contains more than 200,000 single synthesized, well-characterized and drug-like small molecules, and they are easy to obtain. It is a great choice for us to find a lead compound. Meanwhile, an in-house compound library has been established, which is mainly made up of kinase inhibitors. Therefore, the Specs database (221,097 compounds) and in-house compound library (about 3,000 compounds) were combined for the virtual screening. To eliminate compounds with poor drug-like properties, the combined ligand database was employed into DS 2020 and then filtered based on 'Lipinski Rule of Five'[1], 'Veber Rule'[2], and 'Pan Assay Interference Compounds (PAINS)'[3,4]. 'Lipinski Rule of Five' is used to reserve drug-like ligands which have the following features: no more than 5 hydrogen bond donors, no more than 10 hydrogen bond acceptors, molecular weight no more than 500, and LogP no more than 5. The parameter "Number of Violations Allowed" in the 'Lipinski Rule of Five' is 1, which means that the ligands with at least three features mentioned can be reserved. 'Veber Rule' is used to select drug candidates with good oral bioavailability, which have the following features: no more than 10 rotatable bonds, polar surface area no more than 1,400 Å2, or no more than 12 hydrogen bond donors and acceptors. 'PAINS' is used to remove ligands that are not drug-like or lead-like. After the elimination of ligands with poor drug-like properties, 195,483 compounds were reserved, which were then prepared through the *Prepare Ligands* protocol of DS 2020 to add hydrogens, remove duplicates, apply the CHARMM force field and minimize. The prepared ligands can be used for the following virtual screening.

The co-crystal structure of DYRK2 (PDB ID: 6K0J) was downloaded from RCSB Protein Data Bank (http://www.pdb.org)[5], and the disordered conformations, crystal water, and ligand in the co-crystal were removed. The protein was applied to the CHARMM force field and prepared via *Prepare Protein* protocol of DS 2020. The binding site was defined by *From PDB Site Records* protocol for further molecule docking.

The structure-based virtual screening procedure contains two programs, LibDcok and

CDOCKER. LibDock is a molecular docking program based on a high-throughput algorithm, which matches the poses of the ligand according to the structural feature of the receptor[6]. CDOCKER program employs high-temperature molecular dynamics to generate conformation and random rotation of the ligand[7]. Compared with Libdock, CDOCKER is more precise, but it needs more time. Therefore, Libdock was firstly used for primary screening, then CDOCKER was used for further screening.

195,483 prepared compounds firstly docked with DYRK2 protein via the Libdock protocol of DS2020. The pose with the highest score of each ligand was picked out, and ligands ranked based on the 'Libdock Score'. Then 9,696 ligands with a LibDock score of more than 125 were remained, which were further filtered through CDOCKER. Conformation of each ligand was reserved in lowest energy and ligands ranked according to '-CDOCKER INTERACTION ENERGY'. Finally, 2,724 ligands with a "- CDOCKER INTERACTION ENERGY" of more than 55 remained.

Find Diverse Molecules protocol is used to find a diverse subset of ligands from the input ligands. Therefore, to further select ligands from 2,724 ligands, these ligands were clustered into 100 clusters via *Find Diverse Molecules* protocol based on the functional-class fingerprint of diameter 6 (FCFP_6) properties. From 100 clusters, 15 compounds (Supplementary Fig. 3) were selected through visual inspection. In the end, the DYRK2 inhibitory activity of these 15 compounds was evaluated. Among these 15 compounds, compound **12** exhibited the best inhibitory activity against DYRK2 with an IC_{50} value of 263 nM, and IC_{50} values of the other 14 compounds were all more than 10 μM.

Synthesis of DYRK2 inhibitors

Generate synthesis routes. The general synthesis routes of compounds **16**-**27** were described in Supplementary Figure 13. Firstly, intermediates **I-3a-c** were generated through the reductive amination reaction, and **I-5a-d** were synthesized through the condensation reaction. **I-6** reacted with **I-2c** to afford **I-7**, then **I-8** was obtained through the reduction reaction. **I-10a-c** were obtained through the boronation of **I-9a-c**, then coupled with 2,4-dichloro-5-fluoropyrimidine to generate **I-11a-c**. Finally, target compounds **12** and **16**-**24** were synthesized through the Buchwald-Hartwig coupling of

I-11a-c with appropriate amine intermediates. **I-12** reacted with **I-13** in the presence of $Cu(OAc)_2$ to afford **I-14**. Similar to **I-11a-c**, **I-17a** and **I-17b** were generated through boronation reaction and Suzuki coupling. Target compounds **25-27** were also synthesized through the Buchwald-Hartwig coupling reaction.

Characterization of intermediates I-3 - I-21

5-((4-Ethylpiperazin-1-yl)methyl)pyridin-2-amine (I-3a). The mixture of 6-aminonicotinaldehyde **I-1** (0.61 g, 5 mmol) and 1-ethylpiperazine **I-2a** (0.86 g, 7.5 mmol) in 1, 2-dichloroethane (40 mL) was stirred at room temperature for 2 h. Then $NaBH(OAc)_3$ (2.12 g, 10 mmol) was added to the mixture and stirred at room temperature for 8 h. The reaction mixture was quenched by adding 1 M NaOH (50 mL) solution and extracted with DCM (40 mL) for three times. The DCM extract was dried by anhydrous Na_2SO_4 and the solvent was evaporated under reduced pressure. The residue was purified by chromatography on silica to provide **I-3a**, yield 84%. ^{1}H NMR (400 MHz, $CDCl_3$) δ 7.95 (d, J = 2.2 Hz, 1H), 7.45 (dd, J = 8.4, 2.3 Hz, 1H), 6.50 (dd, J = 8.4, 0.8 Hz, 1H), 4.61 (s, 2H), 3.40 (s, 2H), 2.52 – 2.45 (m, 10H), 1.11 (t, J = 7.3 Hz, 3H).

5-((4-Isopropylpiperazin-1-yl)methyl)pyridin-2-amine (I-3b). **I-3b** was synthesized from **I-1** and 1-isopropylpiperazine **I-2b** through the same method with **I-3a**. ^{1}H NMR (400 MHz, $CDCl_3$) δ 7.96 (dd, J = 2.2, 0.8 Hz, 1H), 7.43 (dd, J = 8.4, 2.4 Hz, 1H), 6.49 (dd, J = 8.3, 0.8 Hz, 1H), 4.43 (s, 2H), 3.40 (s, 2H), 2.75 – 2.53 (m, 9H), 1.09 (d, J = 6.5 Hz, 6H).

Tert-butyl* 4-((6-Aminopyridin-3-yl)methyl)piperazine-1-carboxylate *(I-3c). **I-3c** was synthesized from **I-1** and *tert-butyl* piperazine-1-carboxylate **I-2c** through the same method with **I-3a**. ^{1}H NMR (400 MHz, $CDCl_3$) δ 7.95 – 7.94 (m, 1H), 7.44 (dd, J = 8.4, 2.3 Hz, 1H), 6.50 (dd, J = 8.4, 0.8 Hz, 1H), 4.49 (s, 2H), 3.42 (t, J = 5.0 Hz, 4H), 2.37 (t, J = 5.0 Hz, 4H), 1.45 (s, 9H).

(6-Aminopyridin-3-yl)(4-ethylpiperazin-1-yl)methanone (I-5a). The mixture of 6-aminonicotinic acid **I-4a** (0.28 g, 2 mmol) and *N*,*N*'-carbonyldiimidazole (0.39 g, 2.4 mmol) in DMF (5 mL) was stirred at 70 °C for 10 min, then the mixture was stirred at room temperature. After 1 h, 1-ethylpiperazine **I-2a** (0.46 g, 4.0 mmol) was added to the mixture and the mixture was stirred at room temperature overnight. The solvent was evaporated under reduced pressure and the residue was purified by chromatography on silica to provide **I-5a**, yield 81%. ^{1}H NMR (400 MHz, $CDCl_3$) δ 8.17 (dd, J = 2.3, 0.8 Hz, 1H), 7.51 (dd, J = 8.5, 2.3 Hz, 1H), 6.49 (dd, J = 8.5, 0.8 Hz, 1H), 5.16 (s, 2H), 3.71 – 3.63 (m, 4H), 2.48 – 2.43 (m, 6H), 1.10 (t, J = 7.2 Hz, 3H).

(6-Aminopyridin-3-yl)(4-isopropylpiperazin-1-yl)methanone ***(I-5b).*** **I-5b** was synthesized from **I-4a** and 1-isopropylpiperazine **I-2b** through the same method with **I-5a**. ^{1}H NMR (400 MHz, $CDCl_3$) δ 8.18 (dd, J = 2.4, 0.8 Hz, 1H), 7.54 (dd, J = 8.5, 2.3 Hz, 1H), 6.50 (dd, J = 8.5, 0.8 Hz, 1H), 4.83 (s, 2H), 3.69 – 3.61 (m, 4H), 2.78 – 2.70 (m, 1H), 2.56 – 2.52 (m, 4H), 1.05 (d, J = 6.6 Hz, 6H).

Tert-butyl 4-(6-Aminonicotinoyl)piperazine-1-carboxylate (I-5c). **I-5c** was synthesized from **I-4a** and *tert-butyl* piperazine-1-carboxylate **I-2c** through the same method with **I-5a**. ^{1}H NMR (400 MHz, $CDCl_3$) ^{1}H NMR (400 MHz, Chloroform-*d*) δ 8.17 (d, J = 2.4 Hz, 1H), 7.53 (dd, J = 8.4, 2.4 Hz, 1H), 6.51 (d, J = 8.4 Hz, 1H), 4.96 (s, 2H), 3.65 – 3.58 (m, 4H), 3.48 – 3.45 (m, 4H), 1.47 (s, 9H).

(2-Aminopyrimidin-5-yl)(4-ethylpiperazin-1-yl)methanone (I-5d). The mixture of 2-aminopyrimidine-5-carboxylic acid (0.35 g, 2.5 mmol), EDCl (1.5 g, 7.5 mmol), HOBt (0.51 g, 3.75 mmol), and Et_3N (1.25 g, 12.5 mmol) in DMF (20 mL) was stirred for 30 min, then 1-ethylpiperazine **I-2a** (0.31 g, 2.75 mmol) was added to the mixture and the mixture was stirred at room temperature overnight. The solvent was evaporated under reduced pressure and the residue was purified by chromatography on silica to provide **I-5d**, yield 84%. ^{1}H NMR (400 MHz, $CDCl_3$) δ 8.43 (s, 2H), 5.41 (s, 2H), 3.76 – 3.60 (m, 4H), 2.49 – 2.43 (m, 6H), 1.11 (t, J = 7.2 Hz, 3H).

Tert-butyl 4-(6-Nitropyridin-3-yl)piperazine-1-carboxylate (I-7). 5-Bromo-2-nitropyridine **I-6** (0.41 g, 2.0 mmol), *tert-butyl* piperazine-1-carboxylate **I-2c** (0.48 g, 2.6 mmol), and Et_3N (0.41 g, 4.0 mmol) were dissolved in DMSO (5 mL). The reaction mixture was stirred at 70 °C for 18 h. The solvent was evaporated under reduced pressure and the residue was purified by chromatography on silica to provide **I-7**, yield 80%. ^{1}H NMR (400 MHz, $CDCl_3$) δ 8.17 – 8.13 (m, 2H), 7.22 (dd, J = 9.2, 3.1 Hz, 1H), 3.66 – 3.64 (m, 4H), 3.49 – 3.46 (m, 4H), 1.49 (s, 9H).

Tert-butyl 4-(6-Aminopyridin-3-yl)piperazine-1-carboxylate (I-8). **I-7** (0.31 g, 1.0 mmol), Fe powder (0.17 g, 3.0 mmol), and NH_4Cl (0.49 g, 9.0 mmol) were dissolved in 70% EtOH (10 mL). The reaction mixture was stirred at 70 °C for 6 h. The solvent was evaporated under reduced pressure and the residue was purified by chromatography on silica to provide **I-8**, yield 85%. ^{1}H NMR (400 MHz, $CDCl_3$) δ 7.78 (dd, J = 2.9, 0.7 Hz, 1H), 7.17 (dd, J = 8.8, 2.9 Hz, 1H), 6.49 (dd, J = 8.8, 0.7 Hz, 1H), 4.22 (s, 2H), 3.58 – 3.56 (m, 4H), 2.97 – 2.94 (m, 4H), 1.48 (s, 9H).

6-(4,4,5,5-Tetramethyl-1,3,2-dioxaborolan-2-yl)benzo[d]thiazole (I-10a). The mixture of 6-bromobenzo[d]thiazole **I-9a** (0.43 g, 2.0 mmol), bis(pinacolato)diboron (0.53 g, 2.1 mmol), Pd(dppf)Cl_2 (22 mg, 0.06 mmol), and KOAc (0.59 g, 6.0 mmol) in DMF (10 mL) was stirred at 80 °C under Ar atmosphere. After 24 h, the mixture was filtered by diatomite, and the filtrate was evaporated under reduced pressure. The residue was purified by chromatography on silica to afford **I-10a**, yield 90%. ^{1}H NMR (300 MHz, $CDCl_3$) δ 9.07 (s, 1H), 8.46 (s, 1H), 8.14 (d, J = 8.2 Hz, 1H), 7.96 – 7.93 (m, 1H), 1.38 (s, 12H).

2-Methyl-6-(4,4,5,5-tetramethyl-1,3,2-dioxaborolan-2-yl)benzo[d]thiazole (I-10b). **I-10b** was synthesized from 6-bromo-2-methylbenzo[d]thiazole **I-9b** and bis(pinacolato)diboron through the same method with **I-10a**. ^{1}H NMR (400 MHz, $CDCl_3$) δ 8.31 (dd, J = 1.1, 0.7 Hz, 1H), 7.93 (dd, J = 8.1, 0.7 Hz, 1H), 7.86 (dd, J = 8.1, 1.1 Hz, 1H), 2.85 (s, 3H), 1.37 (s, 12H).

6-(4,4,5,5-Tetramethyl-1,3,2-dioxaborolan-2-yl)pyrazolo[1,5-a]pyrimidine (I-10c). **I-10c** was synthesized from 6-bromopyrazolo[1,5-a]pyrimidine **I-9c** and bis(pinacolato)diboron through the same method with **I-10a**. ^{1}H NMR (500 MHz, $CDCl_3$) δ 9.01 – 9.00 (m, 1H), 8.70 – 8.69 (m, 1H), 8.16 – 8.15 (m, 1H), 6.69 – 6.68 (m, 1H), 1.37 (s, 12H).

6-(2-Chloro-5-fluoropyrimidin-4-yl)benzo[d]thiazole (I-11a). The mixture of 2,4-dichloro-5-fluoropyrimidine (0.23 g, 1.4 mmol), $Pd(PPh_3)_2Cl_2$ (21 mg, 0.03 mmol), Na_2CO_3 (0.27 g, 2.5 mmol), DME (10 mL), and H_2O (0.25 mL) were stirred at 80 °C in three-neck flask under Ar atmosphere. **I-10a** (0.26 g, 1.0 mmol) dissolved in the DME (5 mL) was added to the mixture dropwise. After 16 h, the mixture was filtered by diatomite and the filtrate was evaporated under reduced pressure. The residue was purified by chromatography on silica to provide **I-11a**, yield 82%. ^{1}H NMR (400 MHz, $CDCl_3$) δ 9.16 (s, 1H), 8.83 (d, J = 1.7 Hz, 1H), 8.57 (d, J = 3.2 Hz, 1H), 8.34 – 8.25 (m, 2H).

6-(2-Chloro-5-fluoropyrimidin-4-yl)-2-methylbenzo[d]thiazole ***(I-11b).*** **I-11b** was synthesized from **I-10b** and 2,4-dichloro-5-fluoropyrimidine through the same method with **I-11a**. ^{1}H NMR (400 MHz, $CDCl_3$) δ 8.70 (d, J = 1.8 Hz, 1H), 8.55 (d, J = 3.3 Hz, 1H), 8.27 – 8.25 (m, 1H), 8.06 (d, J = 8.7 Hz, 1H), 2.90 (s, 3H).

6-(2-Chloro-5-fluoropyrimidin-4-yl)pyrazolo[1,5-a]pyrimidine (I-11c). **I-11c** was synthesized from **I-10c** and 2,4-dichloro-5-fluoropyrimidine through the same method with **I-11a**. ^{1}H NMR (400 MHz, $CDCl_3$) δ 9.59 (s, 1H), 9.29 (s, 1H), 8.62 (d, J = 2.8 Hz, 1H), 8.30 – 8.29 (m, 1H), 6.84 – 6.83 (m, 1H).

6-Bromo-N,N-dimethylbenzo[d]thiazol-2-amine (I-14). The mixture of 4-bromo-2-iodoaniline **I-12** (0.60 g, 2.0 mmol), sodium dimethyldithiocarbamate **I-13** (0.72 g, 4.0 mmol), $Cu(OAc)_2$ (0.36 g, 2.0 mmol), K_2CO_3 (58 mg, 0.1 mmol) in DMF (10 mL) was

stirred at 120 °C. The mixture was filtered by diatomite after 12 h, and the filtrate was evaporated under reduced pressure. The residue was purified by chromatography on silica to provide **I-14**, yield 85%. ^{1}H NMR (400 MHz, $CDCl_3$) δ 7.69 (d, *J* = 1.9 Hz, 1H), 7.41 – 7.35 (m, 2H), 3.20 (s, 6H).

N,N-Dimethyl-6-(4,4,5,5-tetramethyl-1,3,2-dioxaborolan-2-yl)benzo[d]thiazol-2-amine (I-15). **I-15** was synthesized from **I-14** and bis(pinacolato)diboron through the same method as **I-10a**. ^{1}H NMR (400 MHz, $CDCl_3$) *δ* 8.06 (s, 1H), 7.73 (d, *J* = 7.8 Hz, 1H), 7.54 (d, *J* = 7.8 Hz, 1H), 3.22 (s, 6H), 1.35 (s, 12H).

6-(2-Chloro-5-fluoropyrimidin-4-yl)-N,N-dimethylbenzo[d]thiazol-2-amine (I-17a). **I-17a** was synthesized from **I-15** and 2,4-dichloro-5-fluoropyrimidine **I-16a** through the same method as **I-11a**. ^{1}H NMR (400 MHz, $CDCl_3$) *δ* 8.50 – 8.49 (m, 1H), 8.45 (d, *J* = 3.5 Hz, 1H), 8.17 – 8.14 (m, 1H), 7.62 (d, *J* = 8.7 Hz, 1H), 3.27 (s, 6H).

6-(2-Chlorothieno[3,2-d]pyrimidin-4-yl)-N,N-dimethylbenzo[d]thiazol-2-amine (I-17b). **I-17b** was synthesized from **I-15** and 2,4-dichlorothieno[3,2-d]pyrimidine **I-16b** through the same method as **I-11a**. ^{1}H NMR (300 MHz, $CDCl_3$) *δ* 8.55 (d, *J* = 2.0 Hz, 1H), 8.19 (dd, *J* = 8.5, 2.0 Hz, 1H), 8.08 (d, *J* = 5.5 Hz, 1H), 7.68 (d, *J* = 8.5 Hz, 1H), 7.55 (d, *J* = 5.5 Hz, 1H), 3.28 (s, 6H).

Characterization of Target Compounds 12, 16-27

4-(Benzo[d]thiazol-6-yl)-5-fluoro-N-(5-((4-isopropylpiperazin-1-yl)methyl)pyridin-2-yl)pyrimidin-2-amine (12). The mixture of **I-11a** (133 mg, 0.5 mmol), **I-3b** (141 mg, 0.6 mmol), $Pd_2(dba)_3$ (23 mg, 0.025 mmol), Xantphos (58 mg, 0.1 mmol), Cs_2CO_3 (326 mg, 1.0 mmol), and dioxane (5 mL) was stirred at 100 °C in the sealing tube under Ar atmosphere. The mixture was filtered by diatomite after 12 h, and the filtrate was evaporated under reduced pressure. The residue was purified by chromatography on silica to provide **12** (111 mg, 48%) as a white solid. ^{1}H NMR (400 MHz, $CDCl_3$) *δ* 9.15 (s, 1H), 8.78 (d, *J* = 1.6 Hz, 1H), 8.48 (d, *J* = 3.5 Hz, 1H), 8.38 (dd, *J* = 8.5, 0.8 Hz,

1H), 8.35 – 8.32 (m, 1H), 8.30 – 8.27 (m, 1H), 8.25 – 8.23 (m, 2H), 7.73 (dd, *J* = 8.6, 2.3 Hz, 1H), 3.50 (s, 2H), 2.68 – 2.44 (m, 9H), 1.06 (d, *J* = 6.5 Hz, 6H). HRMS (ESI) for $C_{24}H_{26}FN_7S$ $[M+H]^+$: calcd. 464.2027; found, 464.2016

4-(Benzo[d]thiazol-6-yl)-N-(5-((4-ethylpiperazin-1-yl)methyl)pyridin-2-yl)-5-fluoropyrimidin-2-amine (16). **16** *was* synthesized from **I-11a** and **I-3a** through the same method with **12**. ^{1}H NMR (300 MHz, $CDCl_3$) δ 9.15 (s, 1H), 9.11 (s, 1H), 8.78 – 8.76 (m, 1H), 8.54 (d, *J* = 3.5 Hz, 1H), 8.41 (d, *J* = 8.6 Hz, 1H), 8.35 – 8.26 (m, 3H), 7.73 (dd, *J* = 8.6, 2.3 Hz, 1H), 3.52 (s, 2H), 2.58 – 2.47 (m, 10H), 1.14 (t, *J* = 7.2 Hz, 3H). HRMS (ESI) for $C_{23}H_{24}FN_7S$ $(M + H)^+$: calcd 450.1871; found, 450.1852.

Tert-butyl 4-((6-((4-(Benzo[d]thiazol-6-yl)-5-fluoropyrimidin-2-yl)amino)pyridin-3-yl)methyl)piperazine-1-carboxylate (17). **17** *was* synthesized from **I-11a** and **I-3c** through the same method with **12.** ^{1}H NMR (400 MHz, $CDCl_3$) δ 9.14 (s, 1H), 8.83 (s, 1H), 8.77 (d, *J* = 1.5 Hz, 1H), 8.52 (d, *J* = 3.5 Hz, 1H), 8.41 (d, *J* = 8.6 Hz, 1H), 8.34 – 8.27 (m, 3H), 7.75 – 7.73 (m, 1H), 3.50 (s, 2H), 3.45 – 3.43 (m, 4H), 2.43 – 2.40 (m, 4H), 1.46 (s, 9H). HRMS (ESI) for $C_{26}H_{28}FN_7O_2S$ $(M + H)^+$: calcd 522.2082; found, 522.2074.

4-(Benzo[d]thiazol-6-yl)-5-fluoro-N-(5-(piperazin-1-ylmethyl)pyridin-2-yl)pyrimidin-2-amine hydrogen chloride (18). *Target compound* **17** was dissolved in DCM at 0 °C, then the solution was added HCl gas. After 2 h, the solution was concentrated under reduced pressure to afford **18** without need to purity. ^{1}H NMR (300 MHz, DMSO) δ 12.17 (s, 1H), 10.08 (s, 1H), 9.62 (s, 1H), 8.95 (t, *J* = 2.7 Hz, 2H), 8.71 (s, 1H), 8.53 (d, *J* = 8.8 Hz, 1H), 8.30 (q, *J* = 8.7 Hz, 2H), 7.99 (d, *J* = 9.0 Hz, 1H), 4.57 (s, 2H), 3.54 – 3.42 (m, 8H). HRMS (ESI) for $C_{21}H_{20}FN_7S$ $(M + H)^+$: calcd 422.1558; found, 422.1567.

N-(5-((4-Ethylpiperazin-1-yl)methyl)pyridin-2-yl)-5-fluoro-4-(pyrazolo[1,5-a]pyrimidin-6-yl)pyrimidin-2-amine (19). **19** *was* synthesized from **I-11c** and **I-3a**

through the same method as **12**. ^{1}H NMR (400 MHz, $CDCl_3$) δ 9.52 (dd, J = 2.2, 0.9 Hz, 1H), 9.31 (d, J = 2.2 Hz, 1H), 8.71 (s, 1H), 8.54 (d, J = 3.2 Hz, 1H), 8.31 – 8.28 (m, 2H), 8.27 (d, J = 2.3 Hz, 1H), 7.73 (dd, J = 8.4, 2.4 Hz, 1H), 6.81 (dd, J = 2.2, 0.9 Hz, 1H), 3.52 (s, 2H), 2.57 – 2.46 (m, 10H), 1.12 (t, J = 7.2 Hz, 3H). HRMS (ESI) for $C_{22}H_{24}FN_9$ $(M + H)^+$: calcd 434.2211; found, 434.2216.

(6-((4-(Benzo[d]thiazol-6-yl)-5-fluoropyrimidin-2-yl)amino)pyridin-3-yl)(4-ethylpiperazin-1-yl)methanone (20). **20** *was* synthesized from **I-11a** and **I-5a** through the same method as **12**. ^{1}H NMR (300 MHz, $CDCl_3$) δ 9.68 (s, 1H), 9.15 (s, 1H), 8.76 – 8.75 (m, 1H), 8.61 – 8.58 (m, 2H), 8.52 (dd, J = 8.7, 0.9 Hz, 1H), 8.33 – 8.26 (m, 2H), 7.86 (dd, J = 8.7, 2.3 Hz, 1H), 3.80 – 3.61 (m, 4H), 2.51 – 2.44 (m, 6H), 1.12 (t, J = 7.2 Hz, 3H). HRMS (ESI) for $C_{23}H_{22}FN_7OS$ $(M + H)^+$: calcd 464.1663; found, 464.1657.

4-(Benzo[d]thiazol-6-yl)-5-fluoro-N-(5-(piperazin-1-yl)pyridin-2-yl)pyrimidin-2-amine hydrogen chloride (21). Tert-butyl 4-(6-((4-(benzo[d]thiazol-6-yl)-5-fluoropyrimidin-2-yl)amino)pyridin-3-yl)piperazine-1-carboxylate was firstly synthesized by the reaction of **I-11a** and **I-8** through the same method as **12**, which was further dissolved in DCM at 0 °C. The solution was added HCl gas for 2 h, then concentrated under reduced pressure to afford **21** without need to purity. ^{1}H NMR (400 MHz, DMSO) δ 11.43 (s, 1H), 9.60 (s, 1H), 9.33 (s, 2H), 8.91 (s, 1H), 8.87 (d, J = 3.3 Hz, 1H), 8.33 – 8.23 (m, 2H), 8.07 (d, J = 9.4 Hz, 1H), 8.01 (d, J = 2.9 Hz, 1H), 7.85 (d, J = 9.4 Hz, 1H), 3.44 (t, J = 5.1 Hz, 4H), 3.27 – 3.25 (m, 4H). HRMS (ESI) for $C_{20}H_{18}FN_7S$ $(M + H)^+$: calcd 408.1401; found, 408.1400.

(2-((4-(Benzo[d]thiazol-6-yl)-5-fluoropyrimidin-2-yl)amino)pyrimidin-5-yl)(4-ethylpiperazin-1-yl)methanone (22). **22** *was* synthesized from **I-11a** and **I-5d** through the same method as **12**. ^{1}H NMR (400 MHz, $CDCl_3$) δ 9.15 (s, 1H), 9.10 (s, 1H), 8.85 (d, J = 1.7 Hz, 1H), 8.79 (s, 2H), 8.67 (d, J = 3.4 Hz, 1H), 8.38 – 8.35 (m, 1H), 8.28 (d, J = 8.7 Hz, 1H), 3.81 – 3.63 (m, 4H), 2.53 – 2.49 (m, 6H), 1.13 (t, J = 7.2 Hz, 3H).

HRMS (ESI) for $C_{22}H_{21}FN_8OS$ $(M + H)^+$: calcd 465.1616; found, 465.1608.

(4-Ethylpiperazin-1-yl)(6-((5-fluoro-4-(2-methylbenzo[d]thiazol-6-yl)pyrimidin-2-yl)amino)pyridin-3-yl)methanone (23). **23** was synthesized from **I-11b** and **I-5a** through the same method as **12**. ^1H NMR (400 MHz, $CDCl_3$) δ 9.25 (s, 1H), 8.63 (d, J = 1.8 Hz, 1H), 8.56 – 8.54 (m, 2H), 8.51 (d, J = 8.7 Hz, 1H), 8.24 (dt, J = 8.7, 1.3 Hz, 1H), 8.09 (d, J = 8.6 Hz, 1H), 7.86 (dd, J = 8.7, 2.4 Hz, 1H), 3.78 – 3.64 (m, 4H), 2.90 (s, 3H), 2.51 – 2.46 (m, 6H), 1.13 (t, J = 7.1 Hz, 3H). HRMS (ESI) for $C_{24}H_{24}FN_7OS$ $(M + H)^+$: calcd 478.1820; found, 478.1818.

(6-((5-Fluoro-4-(2-methylbenzo[d]thiazol-6-yl)pyrimidin-2-yl)amino)pyridin-3-yl)(piperazin-1-yl)methanone hydrogen chloride (24). Tert-butyl 4-(6-((5-fluoro-4-(2-methylbenzo[d]thiazol-6-yl)pyrimidin-2-yl)amino)nicotinoyl)piperazine-1-carboxylate was firstly synthesized by the reaction of **I-11b** and **I-5c** through the same method as **12**, which was further dissolved in DCM at 0 °C. The solution was added HCl gas for 2 h, then concentrated under reduced pressure to afford **24** without further purification. ^1H NMR (300 MHz, DMSO) δ 12.04 (s, 1H), 9.85 (s, 2H), 8.92 (d, J = 2.9 Hz, 1H), 8.81 (s, 1H), 8.60 (s, 1H), 8.28 (d, J = 9.1 Hz, 1H), 8.21 (d, J = 8.7 Hz, 1H), 8.13 (d, J = 8.6 Hz, 1H), 8.06 (d, J = 9.0 Hz, 1H), 3.83 – 3.77 (m, 4H), 3.20 – 3.14 (m, 4H), 2.88 (s, 3H). HRMS (ESI) for $C_{22}H_{20}FN_7OS$ $(M + H)^+$: calcd 450.1507;found, 450.1506.

(6-((4-(2-(Dimethylamino)benzo[d]thiazol-6-yl)-5-fluoropyrimidin-2-yl)amino)pyridin-3-yl)(4-isopropylpiperazin-1-yl)methanone(25). **25** *was* synthesized from **I-17a** and **I-5b** through the same method as **12**. ^1H NMR (400 MHz, $CDCl_3$) δ 8.73 (s, 1H), 8.52 – 8.48 (m, 2H), 8.45 – 8.44 (m, 2H), 8.16 – 8.13 (m, 1H), 7.85 (dd, J = 8.7, 2.4 Hz, 1H), 7.65 (d, J = 8.6 Hz, 1H), 3.75 – 3.63 (m, 4H), 3.27 (s, 6H), 2.79 – 2.75 (m, 1H), 2.62 – 2.55 (m, 4H), 1.08 (d, J = 6.5 Hz, 6H). HRMS (ESI) for $C_{26}H_{29}FN_8OS$ $(M + H)^+$: calcd 521.2242; found, 521.2251.

(6-((4-(2-(Dimethylamino)benzo[d]thiazol-6-yl)-5-fluoropyrimidin-2-yl)amino)pyridin-3-yl)(4-isopropylpiperazin-1-yl)methanone (26, YK-2-69). The mixture of **I-17a** (154 mg, 0.5 mmol), **I-5a** (141 mg, 0.6 mmol), $Pd_2(dba)_3$ (23 mg, 0.025 mmol), BINAP (31 mg, 0.05 mmol), sodium tert-butoxide (96 mg, 1.0 mmol), and dioxane (5 mL) was stirred at 100 °C in the sealing tube under Ar atmosphere. The mixture was filtered by diatomite after 12 h, and the filtrate was evaporated under reduced pressure. The residue was purified by chromatography on silica to provide **26** (**YK-2-69**, 139 mg, 55%) as a white solid. ^{1}H NMR (400 MHz, DMSO, Supplementary Fig. 14) δ 10.27 (s, 1H), 8.68 (d, J = 3.8 Hz, 1H), 8.52 (d, J = 2.0 Hz, 1H), 8.35 (d, J = 2.4 Hz, 1H), 8.31 (d, J = 8.7 Hz, 1H), 8.06 (d, J = 8.6 Hz, 1H), 7.86 (dd, J = 8.7, 2.4 Hz, 1H), 7.59 (d, J = 8.6 Hz, 1H), 3.60 – 3.48 (m, 4H), 3.21 (s, 6H), 2.40 – 2.36 (m, 6H), 1.01 (t, J = 7.1 Hz, 3H). ^{13}C NMR (151 MHz, $CDCl_3$, Supplementary Fig. 15) δ 170.6, 168.2, 155.8, 154.9 (d, J = 3.1 Hz), 154.0, 152.0 (d, J = 8.8 Hz), 151.1 (d, J = 256.0 Hz), 147.5, 146.7 (d, J = 27.1 Hz), 137.9, 131.8, 127.3 (d, J = 7.5 Hz), 125.6 (d, J = 5.5 Hz), 124.6, 121.7 (d, J = 7.9 Hz), 118.6, 111.0, 52.2, 40.3, 11.9. HRMS (ESI) for $C_{25}H_{27}FN_8OS$ $(M + H)^+$: calcd 507.2085; found, 507.2097 (Supplementary Fig. 16).

(6-((4-(2-(Dimethylamino)benzo[d]thiazol-6-yl)thieno[3,2-d]pyrimidin-2-yl)amino)pyridin-3-yl)(4-ethylpiperazin-1-yl)methanone (27). **27** was synthesized from **I-17b** and **I-5a** through the same method as **12**. ^{1}H NMR (400 MHz, $CDCl_3$) δ 8.71 (d, J = 8.7 Hz, 1H), 8.56 (s, 1H), 8.48 – 8.47 (m, 2H), 8.17 (dd, J = 8.4, 2.0 Hz, 1H), 7.96 (d, J = 5.5 Hz, 1H), 7.85 (dd, J = 8.8, 2.3 Hz, 1H), 7.69 (d, J = 8.5 Hz, 1H), 7.44 (d, J = 5.5 Hz, 1H), 3.80 – 3.58 (m, 4H), 3.27 (s, 6H), 2.50 – 2.44 (m, 6H), 1.11 (t, J = 7.2 Hz, 3H). HRMS (ESI) for $C_{27}H_{28}N_8OS_2$ $(M + H)^+$: calcd 545.1900; found, 545.1905.

References

1. Lipinski, C.A., Lombardo, F., Dominy, B.W. & Feeney, P.J. Experimental and computational approaches to estimate solubility and permeability in drug discovery and development settings. *Adv. Drug. Deliver. Rev.* **23**, 3-25 (1997).

2. Veber, D.F., et al. Molecular properties that influence the oral bioavailability of drug candidates. *J. Med. Chem.* **45**, 2615-2623 (2002).

3. Walters, W.P. & Murcko, M.A. Prediction of 'drug-likeness'. *Adv. Drug. Deliver. Rev.* **54**, 255-271 (2002).

4. Hann, M., et al. Strategic pooling of compounds for high-throughput screening. *J. Chem. Inf. Comp. Sci.* **39**, 897-902 (1999).

5. Banerjee, S., et al. Ancient drug curcumin impedes 26S proteasome activity by direct inhibition of dual-specificity tyrosine-regulated kinase 2. *Proc. Natl. Acad. Sci. U S A* **115**, 8155-8160 (2018).

6. Diller, D.J. & Merz, K.M. High throughput docking for library design and library prioritization. *Proteins* **43**, 113-124 (2001).

7. Wu, G.S., Robertson, D.H., Brooks, C.L. & Vieth, M. Detailed analysis of grid-based molecular docking: A case study of CDOCKER - A CHARMm-based MD docking algorithm. *J. Comput. Chem.* **24**, 1549-1562 (2003).
