# Supplementary material for: Targeting dual-specificity tyrosine phosphorylation-regulated kinase 2 with a highly selective inhibitor for the treatment of prostate cancer
Source: Nat Commun. 2022 May 25;13:2903. doi: 10.1038/s41467-022-30581-4 (PMC9133015; doi:10.1038/s41467-022-30581-4)
Supplement: Supplementary file 3 — Description to Additional Supplementary Information [file 41467_2022_30581_MOESM3_ESM.pdf]

Description of Additional Supplementary Files

File Name: Supplementary Data 1

Description: Differentially expressed genes between shDYRK2 and YK-2-69 treated samples in transcriptome-wide RNA-sequencing
