# Supplementary material for: Targeting dual-specificity tyrosine phosphorylation-regulated kinase 2 with a highly selective inhibitor for the treatment of prostate cancer
Source: Nat Commun. 2022 May 25;13:2903. doi: 10.1038/s41467-022-30581-4 (PMC9133015; doi:10.1038/s41467-022-30581-4)
Supplement: Supplementary file 5 — Reporting Summary [file 41467_2022_30581_MOESM5_ESM.pdf]

nature portfolio

Corresponding author(s): Peng Yang

Last updated by author(s): Apr 22, 2022

Reporting Summary

Nature Portfolio wishes to improve the reproducibility of the work that we publish. This form provides structure for consistency and transparency in reporting. For further information on Nature Portfolio policies, see our Editorial Policies and the Editorial Policy Checklist.

Statistics

For all statistical analyses, confirm that the following items are present in the figure legend, table legend, main text, or Methods section.

n/a	Confirmed	
☐	☒	The exact sample size (n) for each experimental group/condition, given as a discrete number and unit of measurement
☐	☒	A statement on whether measurements were taken from distinct samples or whether the same sample was measured repeatedly
☐	☒	The statistical test(s) used AND whether they are one- or two-sided<br>*Only common tests should be described solely by name; describe more complex techniques in the Methods section.*
☒	☐	A description of all covariates tested
☒	☐	A description of any assumptions or corrections, such as tests of normality and adjustment for multiple comparisons
☐	☒	A full description of the statistical parameters including central tendency (e.g. means) or other basic estimates (e.g. regression coefficient) AND variation (e.g. standard deviation) or associated estimates of uncertainty (e.g. confidence intervals)
☐	☒	For null hypothesis testing, the test statistic (e.g. F, t, r) with confidence intervals, effect sizes, degrees of freedom and P value noted<br>*Give P values as exact values whenever suitable.*
☒	☐	For Bayesian analysis, information on the choice of priors and Markov chain Monte Carlo settings
☒	☐	For hierarchical and complex designs, identification of the appropriate level for tests and full reporting of outcomes
☒	☐	Estimates of effect sizes (e.g. Cohen's d, Pearson's r), indicating how they were calculated

Our web collection on statistics for biologists contains articles on many of the points above.

Software and code

Policy information about availability of computer code

Data collection	FlowJo V10; GraphPad Prism (8.0); Phoenix WinNonlin (8.3); Discovery Studio (2020); ChemDraw (14.0); Coot (0.8.6.1); PHENIX (1.19.1)
Data analysis	See Methods

For manuscripts utilizing custom algorithms or software that are central to the research but not yet described in published literature, software must be made available to editors and reviewers. We strongly encourage code deposition in a community repository (e.g. GitHub). See the Nature Portfolio guidelines for submitting code & software for further information.

Data

Policy information about availability of data

All manuscripts must include a data availability statement. This statement should provide the following information, where applicable:

- Accession codes, unique identifiers, or web links for publicly available datasets
- A description of any restrictions on data availability
- For clinical datasets or third party data, please ensure that the statement adheres to our policy

The DYRK2-YK-2-69 complex in this study has been deposited in the Protein Data Bank under accession code 7EJV (https://www.rcsb.org/structure/7EJV). The cited DYRK2-EHT 1610, DYRK2-LDN192960 complex in this study can be found in the Protein Data Bank under accession code 5LXD (https://www.rcsb.org/structure/5LXD) and 6K0J (https://www.rcsb.org/structure/6K0J), respectively. The raw RNA-seq data generated in this study have been deposited in the BIG Data Center under the accession number: HRA002197 (https://bigd.big.ac.cn/gsa-human/browse/HRA002197) and HRA002200 (https://bigd.big.ac.cn/gsa-human/browse/HRA002200). Source data are provided with this paper.

Field-specific reporting

Please select the one below that is the best fit for your research. If you are not sure, read the appropriate sections before making your selection.

☒ Life sciences ☐ Behavioural & social sciences ☐ Ecological, evolutionary & environmental sciences

For a reference copy of the document with all sections, see nature.com/documents/nr-reporting-summary-flat.pdf

Life sciences study design

All studies must disclose on these points even when the disclosure is negative.

Sample size	Sample size was determined based on the experimental variability and our experience.
Data exclusions	No data was excluded from the study
Replication	Number of replicates were defined in the figure legends. At least three independent experiments were performed and showed similar results.
Randomization	Group allocation for this study was performed randomly. The same cell passages were used for the biological replicates, and the results were confirmed by different cells passages.
Blinding	For in vivo acute toxicity, pharmacokinetic profiles, and antitumor activity evaluation, the investigators were blinded to group allocation during experiments and outcome assessment. For in vitro experiments, similar results were obtained from at least 3 independent times with 2 independent people.

Reporting for specific materials, systems and methods

We require information from authors about some types of materials, experimental systems and methods used in many studies. Here, indicate whether each material, system or method listed is relevant to your study. If you are not sure if a list item applies to your research, read the appropriate section before selecting a response.

Materials & experimental systems

n/a	Involved in the study	
☐	☒	Antibodies
☐	☒	Eukaryotic cell lines
☒	☐	Palaeontology and archaeology
☐	☒	Animals and other organisms
☐	☒	Human research participants
☒	☐	Clinical data
☒	☐	Dual use research of concern

Methods

n/a	Involved in the study	
☒	☐	ChIP-seq
☐	☒	Flow cytometry
☒	☐	MRI-based neuroimaging

Antibodies

Antibodies used

1.DYRK2 Rabbit Polyclonal Antibody (Abcepta, Cat#AP7534a, Lot#SA120428AI, 1:1000)
2.RB Rabbit Polyclonal Antibody (proteintech, Cat#10048-2-Ig, 1:5000)
3.Phospho-Rb (Ser807/811) Rabbit Monoclonal Antibody (Cell Signaling Technology, Cat#8516, clone name: D20B12, 1:1000)
4.CDK4 Rabbit Monoclonal Antibody (Cell Signaling Technology, Cat#12790, clone name: D9G3E, 1:1000)
5.CDK6 Mouse Monoclonal Antibody (Cell Signaling Technology, Cat#3136, clone name: DCS83, 1:2000)
6.PARP Rabbit Monoclonal Antibody (Cell Signaling Technology, Cat#9532, clone name: 46D11, 1:1000)
7.Cleaved PARP Rabbit Monoclonal Antibody (Beyotime, Cat#AF1567, clone name: P09874, 1:1000)
8.RRS1 Rabbit Polyclonal Antibody (Proteintech, Cat#15329-1-AP, 1:1000)
9.CCNG2 Rabbit Polyclonal Antibody (Abcam, Cat#ab251826, 1:500)
10.P21 Rabbit Polyclonal Antibody (Proteintech, Cat#10355-1-AP, 1:1000)
11.P27 Rabbit Monoclonal Antibody (Cell Signaling Technology, Cat#3686, clone name: D69C12, 1:1000)
12.P53 Rabbit Monoclonal Antibody (Cell Signaling Technology, Cat#2527, clone name: 7F5, 1:1000)
13.XIAP Rabbit Polyclonal Antibody (Proteintech, Cat#10037-1-Ig, 1:1000)
14.E-Cadherin Rabbit Polyclonal Antibody (Proteintech, Cat#20874-1-AP, 1:5000)
15.4E-BP1 Rabbit Monoclonal Antibody (Cell Signaling Technology, Cat#9644, Lot#12, clone name: 53H11, 1:1000)
16.Phospho-4E-BP1 (Thr37/46) Rabbit Monoclonal Antibody (Cell Signaling Technology, Cat#2855, Lot#26, clone name: 236B4, 1:1000)
17.Vinculin Rabbit Polyclonal Antibody (Proteintech, Cat#26520-1-AP, 1:1000)
18.Alpha Tubulin Mouse Monoclonal Antibody (Proteintech, Cat#66031-1-Ig, clone name: 1E4C11, 1:20000)
19.Goat Anti-Mouse IgG, H&L Chain Specific Peroxidase Conjugate,(Merck, Cat#401215-2ML, 1:5000
20.Goat Anti-Rabbit IgG, H & L Chain Specific Peroxidase Conjugate Merck, Cat#401315-2ML, 1:5000
21.Rabbit IgG(Beyotime, Cat#A7016, 1:200)

Validation	1. DYRK2 Rabbit Polyclonal Antibody detects endogenous levels of total DYRK2 protein; Applications: Immunohistochemistry (IHC), Immunoprecipitation (IP), and Western Blot (WB). Validation Refs. from manufacturer's website: https://www.abcepta.com.cn/products/AP7534a-DYRK2-Antibody-N-term<br>2. RB Rabbit Polyclonal Antibody detects endogenous levels of total Rb protein (both phosphorylated and unphosphorylated forms); Applications:WB. Validation Refs. from manufacturer's website: https://www.ptgcn.com/products/RB1-Antibody-10048-2-Ig.htm<br>3. Phospho-Rb (Ser807/811) Rabbit Monoclonal Antibody detects endogenous levels of Rb protein only when phosphorylated at Ser807/811; Applications:WB. Validation Refs. from manufacturer's website: https://www.cellsignal.com/products/primary-antibodies/phospho-rb-ser807-811-d20b12-xp-rabbit-mab/8516<br>4. CDK4 Rabbit Monoclonal Antibody detects endogenous levels of total CDK4 protein; Application: WB. Validation Refs. from manufacturer's website: https://www.cellsignal.com/products/primary-antibodies/cdk4-d9g3e-rabbit-mab/12790<br>5. CDK6 Mouse Monoclonal Antibody detects endogenous levels of total CDK6 protein; Application: WB. Validation Refs. from manufacturer's website: https://www.cellsignal.com/products/primary-antibodies/cdk6-dcs83-mouse-mab/3136<br>6.PARP Rabbit Monoclonal Antibody recognizes full length PARP1; Applications: WB. Validation Refs. from manufacturer's website:https://www.cellsignal.com/products/primary-antibodies/parp-46d11-rabbit-mab/9532<br>7. Cleaved PARP Rabbit Monoclonal Antibody recognizes large fragment of human PARP1 produced by caspase cleavage; Applications: WB. Validation Refs. from manufacturer's website:https://beyotime.com/product/AF1567.htm<br>8.RRS1 Rabbit Polyclonal Antibody detects endogenous levels of total RRS1 protein; Application: WB. Validation Refs. from manufacturer's website: https://www.ptgcn.com/products/RRS1-Antibody-15329-1-AP.htm<br>9. CCNG2 Rabbit Polyclonal Antibody detects endogenous levels of total CCNG2 protein; Application: WB. Validation Refs. from manufacturer's website: https://www.abcam.cn/ccng2-antibody-ab251826.html<br>10. P21 Rabbit Polyclonal Antibody detects endogenous levels of total P21 protein; Application: WB. Validation Refs. from manufacturer's website: https://www.ptgcn.com/products/P21-Antibody-10355-1-AP.htm<br>11. P27 Rabbit Monoclonal Antibody detects endogenous levels of total P27 protein; Application: WB. Validation Refs. from manufacturer's website: https://www.cellsignal.cn/products/primary-antibodies/p27-kip1-d69c12-xp-rabbit-mab/3686<br>12. P53 Rabbit Monoclonal Antibody detects endogenous levels of total P53 protein; Application: WB. Validation Refs. from manufacturer's website: https://www.cellsignal.cn/products/primary-antibodies/p53-7f5-rabbit-mab/2527<br>13. XIAP Rabbit Polyclonal Antibody detects endogenous levels of total XIAP protein; Application: WB. Validation Refs. from manufacturer's website: https://www.ptgcn.com/products/XIAP-Antibody-10037-1-Ig.htm<br>14. E-Cadherin Rabbit Polyclonal Antibody detects endogenous levels of total E-cadherin protein; Application: WB. Validation Refs. from manufacturer's website: https://www.ptgcn.com/products/E-cadherin-Antibody-20874-1-AP.htm<br>15. 4E-BP1 Rabbit Monoclonal Antibody detects endogenous levels of total 4E-BP1 protein (both phosphorylated and unphosphorylated forms);; Application: WB. Validation Refs. from manufacturer's website: https://www.cellsignal.cn/products/primary-antibodies/4e-bp1-53h11-rabbit-mab/9644<br>16. Phospho-4E-BP1 (Thr37/46) Rabbit Monoclonal Antibody detects endogenous levels of Rb protein only when phosphorylated at Thr37/46; Applications:WB. Validation Refs. from manufacturer's website: https://www.cellsignal.cn/products/primary-antibodies/phospho-4e-bp1-thr37-46-236b4-rabbit-mab/2855<br>17.Vinculin Rabbit Polyclonal Antibody is used as the control in the WB. Validation Refs. from manufacturer's website: https://www.ptgcn.com/products/Vinculin-Antibody-26520-1-AP.htm<br>18.Alpha Tubulin Mouse Monoclonal Antibody is used as the control in the WB. Validation Refs. from manufacturer's website:https://www.ptgcn.com/products/tubulin-Alpha-Antibody-66031-1-Ig.htm<br>19.Goat Anti-Mouse IgG, H&L Chain Specific Peroxidase Conjugate is used as the secondary antibody in the WB. Validation Refs. from manufacturer's website: https://www.sigmaaldrich.cn/CN/zh/product/mm/401215<br>20.Goat Anti-Rabbit IgG, H & L Chain Specific Peroxidase Conjugate is used as the secondary antibody in the WB. Validation Refs. from manufacturer's website: https://www.sigmaaldrich.cn/CN/zh/product/mm/401315m<br>21.Rabbit IgG is used as the control in the IP. Validation Refs. from manufacturer's website: https://beyotime.com/product/A7016.htm

Eukaryotic cell lines

Policy information about cell lines

Cell line source(s)	DU145 (ATCC), PC-3 (ATCC), 22Rv1 (ATCC), LNcaP (ATCC), RWPE-1 (ATCC), 293T (ATCC)
Authentication	Cell lines were authenticated by STR by KeyGEN BioTECH.Co.Ltd and using PCR assays with species-specific primers.
Mycoplasma contamination	All cell lines were tested negative for mycoplasma contamination
Commonly misidentified lines (See ICLAC register)	None of the cell lines used in this study are commonly misidentified.

Animals and other organisms

Policy information about studies involving animals; ARRIVE guidelines recommended for reporting animal research

Laboratory animals	For acute toxicity studies, ICR mice were used, seven week old, half male and half female;<br>For pharmacokinetic profiles measurement, SD rats were used, 6-8 week old, male;<br>For in vivo antitumor activity evaluation, BALB/c nude mice were used, 4-5 week old, male;<br>The temperature and humidity of the animal room is 20 - 26 ºC and 40 - 70%, respectively. All animals were given 12 h of light and 12 h of darkness in turn each day.
Wild animals	No wild animals were used in this study.
Field-collected samples	No field-collected samples were used in this study.

Ethics oversight | The experimental procedures and animal use and care protocols in the acute toxicity, pharmacokinetic profiles, and in vivo antitumor activity evaluation of this study were approved by the Institutional Animal Care and Use Committee (IACUC) of China Pharmaceutical University.

Note that full information on the approval of the study protocol must also be provided in the manuscript.

Human research participants

Policy information about studies involving human research participants

Population characteristics | Three pairs of prostate tumor and matched normal tissues were obtained from Huai'an First People's Hospital with patients' informed consent.Clinicopathologic information of these three patient was listed in Supplementary Table S3

Recruitment | Selection of prostate cancer patients was based on availability, without any particular standard

Ethics oversight | The human samples used in this study were approved by Ethics Committee of Huai'an First People's Hospital Ethics Committee.

Note that full information on the approval of the study protocol must also be provided in the manuscript.

Flow Cytometry

Plots

Confirm that:

☒ The axis labels state the marker and fluorochrome used (e.g. CD4-FITC).

☒ The axis scales are clearly visible. Include numbers along axes only for bottom left plot of group (a 'group' is an analysis of identical markers).

☒ All plots are contour plots with outliers or pseudocolor plots.

☒ A numerical value for number of cells or percentage (with statistics) is provided.

Methodology

Sample preparation | For cell cycle analysis, cells were collected and fixed with 70% ethanol overnight at 4 °C followed by staining propidium iodide (PI) using Cell Cycle Analysis Kit (Beyotime, C1052) for 30 min at 37 °C.
For cell apoptosis analysis, Annexin V-APC and PI (Elabscience, E-CK-A217) was used. Cells were collected and stained with 5 µL Annexin V-APC and PI, for 20 min at room temperature away from light.

Instrument | FACS Celesta (BD)

Software | FlowJo V10 software

Cell population abundance | No cell sorting was done.

Gating strategy | All gates were set based on negative control and isotype control after appropriate compensation adjustment.

☒ Tick this box to confirm that a figure exemplifying the gating strategy is provided in the Supplementary Information.
